# T cell receptor repertoires associated with control and disease progression following *Mycobacterium tuberculosis* infection

**DOI:** 10.1038/s41591-022-02110-9

**Published:** 2023-01-05

**Authors:** Munyaradzi Musvosvi, Huang Huang, Chunlin Wang, Qiong Xia, Virginie Rozot, Akshaya Krishnan, Peter Acs, Abhilasha Cheruku, Gerlinde Obermoser, Alasdair Leslie, Samuel M. Behar, Willem A. Hanekom, Nicole Bilek, Michelle Fisher, Stefan H. E. Kaufmann, Gerhard Walzl, Mark Hatherill, Mark M. Davis, Thomas J. Scriba, Adolescent Cohort Study team, Fazlin Kafaar, Leslie Workman, Humphrey Mulenga, Thomas J. Scriba, E. Jane Hughes, Nicole Bilek, Mzwandile Erasmus, Onke Nombida, Ashley Veldsman, Yolundi Cloete, Deborah Abrahams, Sizulu Moyo, Sebastian Gelderbloem, Michele Tameris, Hennie Geldenhuys, Willem Hanekom, Gregory Hussey, Rodney Ehrlich, Suzanne Verver, Larry Geiter, GC6-74 Consortium, Gerhard Walzl, Gillian F. Black, Gian van der Spuy, Kim Stanley, Magdalena Kriel, Nelita Du Plessis, Nonhlanhla Nene, Teri Roberts, Leanie Kleynhans, Andrea Gutschmidt, Bronwyn Smith, Andre G. Loxton, Novel N. Chegou, Gerhardus Tromp, David Tabb, Tom H. M. Ottenhoff, Michel R. Klein, Marielle C. Haks, Kees L. M. C. Franken, Annemieke Geluk, Krista E. van Meijgaarden, Simone A. Joosten, W. Henry Boom, Bonnie Thiel, Harriet Mayanja-Kizza, Moses Joloba, Sarah Zalwango, Mary Nsereko, Brenda Okwera, Hussein Kisingo, Stefan H. E. Kaufmann, Shreemanta K. Parida, Robert Golinski, Jeroen Maertzdorf, January Weiner, Marc Jacobson, Hazel M. Dockrell, Maeve Lalor, Steven Smith, Patricia Gorak-Stolinska, Yun-Gyoung Hur, Ji-Sook Lee, Amelia C. Crampin, Neil French, Bagrey Ngwira, Anne Ben-Smith, Kate Watkins, Lyn Ambrose, Felanji Simukonda, Hazzie Mvula, Femia Chilongo, Jacky Saul, Keith Branson, Sara Suliman, Thomas J. Scriba, Hassan Mahomed, E. Jane Hughes, Nicole Bilek, Mzwandile Erasmus, Onke Nombida, Ashley Veldsman, Katrina Downing, Michelle Fisher, Adam Penn-Nicholson, Humphrey Mulenga, Brian Abel, Mark Bowmaker, Benjamin Kagina, William Kwong Chung, Willem A. Hanekom, Jerry Sadoff, Donata Sizemore, S. Ramachandran, Lew Barker, Michael Brennan, Frank Weichold, Stefanie Muller, Larry Geiter, Desta Kassa, Almaz Abebe, Tsehayenesh Mesele, Belete Tegbaru, Debbie van Baarle, Frank Miedema, Rawleigh Howe, Adane Mihret, Abraham Aseffa, Yonas Bekele, Rachel Iwnetu, Mesfin Tafesse, Lawrence Yamuah, Martin Ota, Jayne Sutherland, Philip Hill, Richard Adegbola, Tumani Corrah, Martin Antonio, Toyin Togun, Ifedayo Adetifa, Simon Donkor, Peter Andersen, Ida Rosenkrands, Mark Doherty, Karin Weldingh, Gary Schoolnik, Gregory Dolganov, Tran Van

**Affiliations:** 1 South African Tuberculosis Vaccine Initiative, Institute of Infectious Disease and Molecular Medicine, and Division of Immunology, Department of Pathology, University of Cape Town, Cape Town, South Africa; 2 Institute for Immunity, Transplantation and Infection, Stanford University School of Medicine, Stanford, CA USA; 3 Human Immune Monitoring Center, Stanford University, Stanford, CA USA; 4 Africa Health Research Institute, Durban, South Africa; 5 School of Laboratory Medicine and Medical Sciences, College of Health Sciences, University of KwaZulu-Natal, Durban, South Africa; 6 Department of Infection and Immunity, University College London, London, UK; 7 Department of Microbiology and Physiological Systems, University of Massachusetts Chan Medical School, Worcester, MA USA; 8 Max Planck Institute for Infection Biology, Berlin, Germany; 9 Max Planck Institute for Multidisciplinary Sciences, Göttingen, Germany; 10 Hagler Institute for Advanced Study, Texas A&M University, College Station, TX USA; 11 DST-NRF Centre of Excellence for Biomedical Tuberculosis Research, South African Medical Research Council Centre for Tuberculosis Research; Division of Molecular Biology and Human Genetics, Faculty of Medicine and Health Sciences, Stellenbosch University, Cape Town, South Africa; 12 Howard Hughes Medical Institute, Stanford University, Stanford, CA USA; 13 Department of Microbiology and Immunology, Stanford University School of Medicine, Stanford, CA USA; 14 South African Tuberculosis Vaccine Initiative, Institute of Infectious Disease and Molecular Medicine and Division of Immunology, Department of Pathology, University of Cape Town, Cape Town, South Africa; 15 School of Public Health and Family Medicine, University of Cape Town, Cape Town, South Africa; 16 KNCV Tuberculosis Foundation The Hague Amsterdam Institute of Global Health and Development, Academic Medical Centre, Amsterdam, the Netherlands; 17 Aeras, Rockville, MA USA; 18 DST-NRF Centre of Excellence for Biomedical TB Research and MRC Centre for TB Research, Division of Molecular Biology and Human Genetics, Faculty of Medicine and Health Sciences, Stellenbosch University, Tygerberg, South Africa; 19 Department of Infectious Diseases, Leiden University Medical Centre, Leiden, the Netherlands; 20 Tuberculosis Research Unit, Department of Medicine, Case Western Reserve University School of Medicine and University Hospitals Case Medical Center, Cleveland, OH USA; 21 Department of Medicine and Department of Microbiology, College of Health Sciences, Faculty of Medicine, Makerere University, Kampala, Uganda; 22 Department of Immunology, Max Planck Institute for Infection Biology, Berlin, Germany; 23 Department of Immunology and Infection, Faculty of Infectious and Tropical Diseases, London School of Hygiene and Tropical Medicine, London, UK; 24 Karonga Prevention Study, Chilumba, Malawi; 25 South African Tuberculosis Vaccine Initiative, Institute of Infectious Disease and Molecular Medicine and Division of Immunology, Department of Pathology, University of Cape Town, Cape Town, South Africa; 26 Ethiopian Health and Nutrition Research Institute, Addis Ababa, Ethiopia; 27 University Medical Centre, Utrecht, the Netherlands; 28 Armauer Hansen Research Institute, Addis Ababa, Ethiopia; 29 Vaccines and Immunity Theme, Medical Research Council Unit, Fajara, The Gambia; 30 Department of Infectious Disease Immunology, Statens Serum Institute, Copenhagen, Denmark; 31 Department of Microbiology and Immunology, Stanford University, Stanford, CA USA

**Keywords:** Tuberculosis, Immunological surveillance, Infection, T-cell receptor

## Abstract

Antigen-specific, MHC-restricted αβ T cells are necessary for protective immunity against *Mycobacterium tuberculosis*, but the ability to broadly study these responses has been limited. In the present study, we used single-cell and bulk T cell receptor (TCR) sequencing and the GLIPH2 algorithm to analyze *M. tuberculosis*-specific sequences in two longitudinal cohorts, comprising 166 individuals with *M. tuberculosis* infection who progressed to either tuberculosis (*n* = 48) or controlled infection (*n* = 118). We found 24 T cell groups with similar TCR-β sequences, predicted by GLIPH2 to have common TCR specificities, which were associated with control of infection (*n* = 17), and others that were associated with progression to disease (*n* = 7). Using a genome-wide *M. tuberculosis* antigen screen, we identified peptides targeted by T cell similarity groups enriched either in controllers or in progressors. We propose that antigens recognized by T cell similarity groups associated with control of infection can be considered as high-priority targets for future vaccine development.

## Abstract (web summary)

Analysis of peripheral mycobacteria-reactive CD4^+^ T cell receptor sequences from individuals infected with *Mycobacterium* *tuberculosis* shows a high degree of overlap between progressors and controllers, but points to some distinct clonotypes that are enriched in either group.

## Main

Antigen-specific CD4 T cells are necessary for protective immunity against *M. tuberculosis*, the etiological agent of tuberculosis (TB)^1,2^. Experimental and clinical evidence shows that the primary T cell mediators of this protection are interferon (IFN)-γ-expressing helper type 1 T cells (T_H_1 cells), although recent evidence from nonhuman primates implicates T_H_1/T_H_17 cells as probable correlates of protection^3–6^. Comprehensive delineation of αβ T cell responses in *M. tuberculosis*-infected humans has been hampered by the complexity and heterogeneity of clinical phenotypes in TB^7,8^, the high interindividual diversity of the major histocompatibility complex (MHC), which restricts antigen presentation to T cells, and the marked diversity of TCRs^9,10^, even within single hosts.

Recent advances in single-cell and bulk TCR-sequencing technologies enable characterization of the antigen-specific TCR repertoire with unprecedented throughput and efficiency^11,12^. In addition, advances in analytic approaches, particularly GLIPH^12^ and GLIPH2 (ref. 13), allow grouping of TCR sequences that share conserved sequences and motifs in the CDR3 region, which is primarily responsible for the recognition of antigenic peptides bound to molecules of the MHC^9,14,15^. This allows rapid clustering of thousands or millions of TCRs into similarity groups, without having to know for what antigens these TCRs are specific. This enables a broad profiling of T cell specificities, despite the complexity of these responses across individuals and groups^13,15,16^. Together, these tools provide the opportunity to analyze the pathogen-specific T cell response in a holistic and unbiased manner that was not previously possible.

We hypothesized that distinct *M. tuberculosis*-specific T cell clonotype groups in *M. tuberculosis*-infected individuals are associated with either protection against or risk of disease progression. We applied antigen-specific T cell repertoire profiling and analyses to two well-characterized, longitudinal cohorts of *M. tuberculosis*-infected individuals, some of whom successfully controlled infection (controllers) and others who progressed to TB disease (progressors). We identified mycobacteria-reactive T cell groups with similar TCRs (similarity groups, which probably recognize the same epitope) and compared their frequencies in *M. tuberculosis*-infected controllers and progressors, to define putative protective (enriched in controllers) or pathogenic or nonprotective (enriched in progressors) TCR similarity groups. We then identified the *M. tuberculosis* antigenic epitope and restricted MHC for a subset of TCR members of such similarity groups using genome-wide antigen screening. Particularly important in this respect is that we were able to identify a set of controller-associated *M. tuberculosis* antigens that may be excellent candidates for inclusion in a future TB vaccine.

## Results

### Defining *M. tuberculosis*-specific T cells and their repertoires

**Fig. 1:**
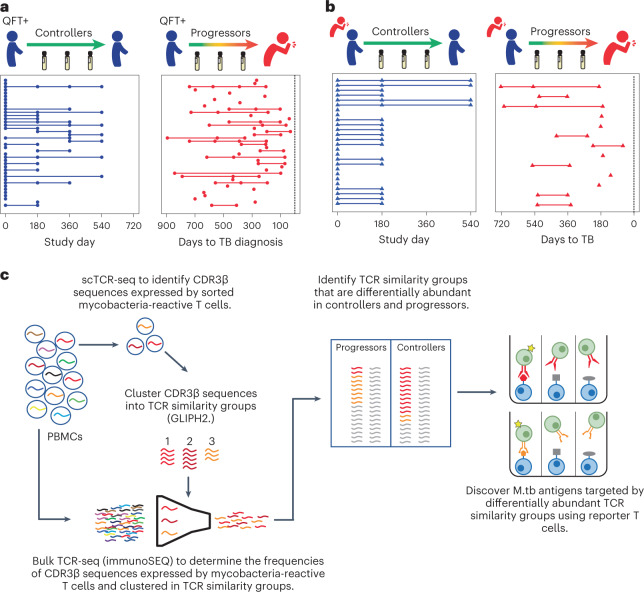
Identification of TCR sequences and antigens recognized by *M. tuberculosis* lysate-responsive T cells in controllers and progressors. **a**,**b**, Plots depicting longitudinal study timepoints (dots) at which PBMC samples were analyzed for each individual controller (blue) or progressor (red, synchronized to TB diagnosis) in the ACS (**a**) or the GC6-74 cohort (**b**). Each horizontal line or symbol represents an individual. **c**, Experimental workflow and analysis approach used to identify mycobacteria-reactive CDR3αβ sequences and determine their frequencies. First, scTCR-seq was performed on sorted mycobacteria-reactive T cells expressing the activation markers CD69 and CD154 or CD137 after in vitro *M. tuberculosis* (M.tb) lysate stimulation. GLIPH2 analysis clustered TCR sequences expressed by mycobacteria-reactive T cells into TCR similarity groups. In parallel, bulk TCR-seq was performed on PMBCs (unstimulated) to profile the repertoire and determine the frequencies of CDR3β sequences in each sample. The total frequencies of CDR3β sequences within a GLIPH2 TCR similarity group were determined for each controller and progressor sample using the bulk TCR-seq data. For controllers and progressors with samples collected at multiple study timepoints, the total frequencies of CDR3β sequences within a TCR similarity group were determined for each timepoint. The total frequencies of CDR3β sequences within a TCR similarity group were compared in controllers and progressors. To identify antigens recognized by these antigen-specific T cells, transduced NFAT, reporter stable J76-NFATRE-luc T cell line cells expressing representative TCR-αβ chains from TCR similarity groups found to be differentially abundant in controllers and progressors were coincubated with aAPCs to screen the *M. tuberculosis* proteome. QFT, QuantiFERON-TB Gold.

**Fig. 2:**
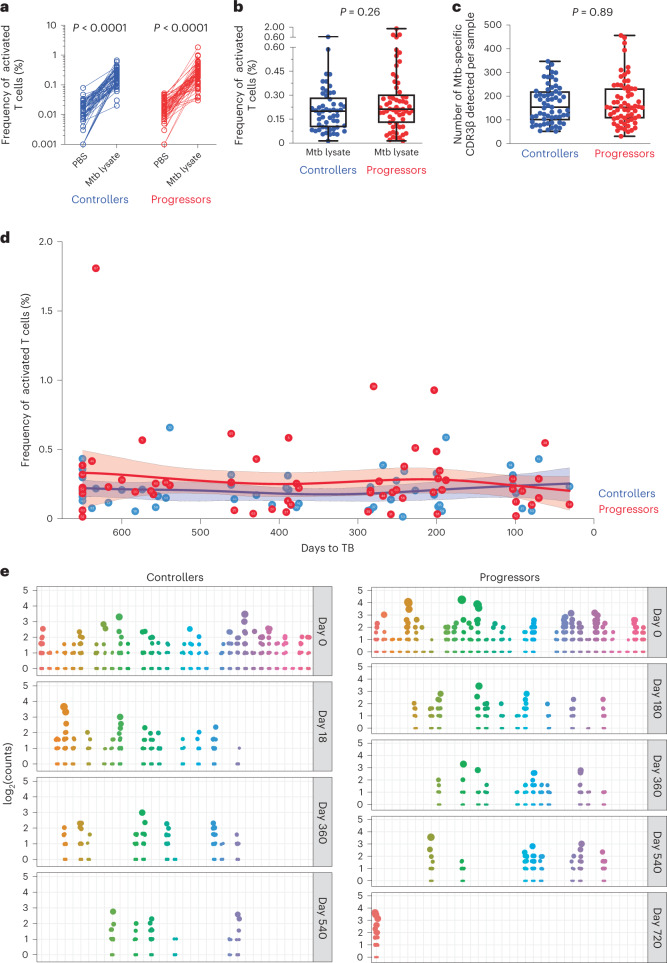
Similar frequencies and counts of *M. tuberculosis* lysate-reactive T cells in controllers and progressors. **a**, Plot showing the frequencies of T cells coexpressing CD69 and CD154 or CD69 and CD137 (activated T cells), measured by flow cytometry after PBS (negative control) or *M. tuberculosis* (Mtb) lysate stimulation. Each dot represents an individual sample (controllers, *n* = 61; progressors, *n* = 64). **b**, A plot depicting the background subtracted frequencies of activated T cells. The horizontal lines represent medians, the bounds of the boxes indicate the 25th and 75th percentiles and the whiskers represent the minima and maxima. Each dot represents an individual sample (controllers, *n* = 53; progressors, *n* = 61). The *P* value was calculated using the Mann–Whitney *U*-test (two sided). Note that some samples are from the same participant collected at different study timepoints. **c**, A plot depicting the numbers of detected CDR3β sequences from sorted, *M. tuberculosis*-specific T cells identified by TCR-seq in PBMCs from controllers and progressors in the ACS cohort. Each dot represents an individual sample (controllers, *n* = 61; progressors, *n* = 64). The horizontal lines represent medians, the bounds of the boxes indicate the 25th and 75th percentiles and the whiskers represent the minima and maxima. The *P* value was calculated using the Mann–Whitney *U*-test (two sided). Some samples are from the same participants collected at different study timepoints. **d**, Plot depicting the kinetics (background subtracted) of *M. tuberculosis* lysate-reactive T cells, measured by flow cytometry, after PBMC stimulation with *M. tuberculosis* lysate in controllers and progressors from the ACS cohort. Progressor samples were synchronized according to their time to TB diagnosis and controller samples were synchronized to their matched progressors. The solid lines indicate the modeled nonlinear splines and the shaded bands represent 95% CIs. **e**, Plots depicting clonal expansions of *M. tuberculosis* lysate-reactive T cells in samples from controllers and progressors at different timepoints (in days) after enrollment. Each dot represents a unique CDR3β sequence observed in a sample. The size of the dot is relative to the number of times the sequence was detected. Plots have been aligned by participant on the horizontal axis.

**Extended Data Fig. 1:**
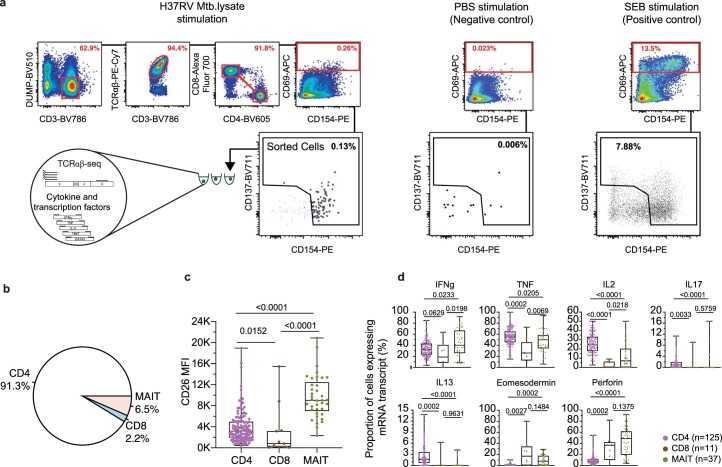
CD4 T cells are the predominant responding T cell subset to M.tb lysate stimulation. (**a**) Representative flow cytometry plots depicting the gating strategy used to identify and sort activated M.tb lysate-reactive T cells in PBMC stimulated with M.tb lysate. M.tb-specific T cells were defined as live CD3+, TCRαβ+ CD4+ or CD8+ T cells that co-express CD69 and CD154 or CD69 and CD137. Note the value in each plot is the percentage of parent (red) or grandparent (black). Plots depict data from M.tb lysate stimulation, PBS (negative control) and SEB (positive control) stimulations. (**b**) Pie chart depicting the proportions of sorted single T cells that responded to M.tb lysate stimulation by co-expressing CD69 and CD154 or CD69 and CD137, by CD4, CD8 or MAIT cell subset. (MAIT cells were defined as expressing canonical MAIT CDR3α sequences, determined using MAIT Match50). (**c**) Expression levels of CD26, measured as median fluorescent intensity (MFI), on sorted CD4, CD8 or MAIT cells from ACS controllers and progressors. The horizontal lines represent medians, the bounds of the boxes indicate the 25% and 75% percentile and the whiskers represent the minima and maxima. Each dot represents an individual sample and only samples with ≥10 cells were included. (**d**) Proportions of CD4, CD8 or MAIT cells that express the indicated mRNA transcripts in sorted T cells from ACS controllers and progressors. The horizontal lines represent medians, the bounds of the boxes indicate the 25% and 75% percentile and the whiskers represent the minima and maxima. Each dot represents an individual sample and only samples with ≥10 cells were included. A threshold of ≥5 transcript reads per cell was used to classify a cell as positive for the indicated transcript. P-value were calculated with the Mann-Whitney U test (two-sided).

We first determined TCR-αβ sequences expressed by mycobacteria-reactive T cells in controllers and progressors selected from adolescents with evidence of *M. tuberculosis* infection who participated in the Adolescent Cohort Study (ACS), a large epidemiological study of TB^17^. Progressors (*n* = 44) developed microbiologically confirmed, intrathoracic TB over 2 years of follow-up. Controllers (*n* = 44) also had evidence of *M. tuberculosis* infection, but did not develop TB during follow-up. Mycobacteria-reactive T cells were identified by stimulating thawed peripheral blood mononuclear cells (PBMCs) from progressors and controllers with *M. tuberculosis* lysate, comprising both protein and nonprotein antigens, and sorting activated CD4 or CD8 T cells (Fig. 1a and Extended Data Fig. 1a). Activated T cells were identified by their elevated expression levels of CD69 together with CD154 or CD137 for single-cell TCR-sequencing (scTCR-seq). We successfully captured the TCR-αβ repertoire of *M. tuberculosis*-lysate-responsive T cells from PBMC samples collected from 35 controllers and 35 progressors using this scTCR-seq approach (Supplementary Table 1). Among 37,674 sorted T cells from progressors and controllers, 22,276 (59.1%) CDR3α and 21,404 (56.8%) CDR3β sequences were detected, of which 15,272 and 16,517 were unique, respectively (Supplementary Table 2). Higher frequencies of activated T cells were observed after stimulation with *M. tuberculosis* lysate compared with phosphate-buffered saline (PBS), but frequencies of activated T cells between controllers and progressors were not different, nor were the numbers of CDR3β sequences detected (Fig. 2a–c). In addition, frequencies of activated T cells were constant over the 2-year follow-up period (Fig. 2d). Clonal expansions (two or more clones) were observed in scTCR data from all but four samples (Fig. 2e). More than 90% of sorted *M. tuberculosis* lysate-reactive T cells were CD4 T cells, 2.2% were CD8 T cells and 6.5% expressed canonical mucosa-associated invariant T (MAIT) cell CDR3α sequences irrespective of CD4 and CD8 expression (Extended Data Fig. 1b). These results are consistent with previous studies which showed that *M. tuberculosis*-reactive T cells are predominately CD4 T cells^13,15^. Cells expressing known canonical MAIT CDR3α sequences expressed markedly higher levels of CD26, a marker associated with MAIT cells^18^, compared with CD4 and CD8 T cells (Extended Data Fig. 1c), demonstrating that the phenotype of single-cell sorted cells faithfully aligns with the TCR identity. Expected levels of messenger RNA expression of known functional markers by sorted CD4, CD8 and MAIT cells further validated the experimental TCR-seq pipeline we used. For example, a higher proportion of *M. tuberculosis* lysate-responsive MAIT cells expressed IFN-γ mRNA transcripts compared with CD4 and CD8 T cells, whereas a higher proportion of CD4 T cells expressed tumor necrosis factor (TNF), interleukin (IL)-2, IL-17 and IL-13 mRNA transcripts than CD8 and MAIT cells, and higher proportions of CD8 T cells and MAIT cells expressed eomesodermin and perforin mRNA transcripts than CD4 T cells (Extended Data Fig. 1d).

### Comparison of *M. tuberculosis* TCR groups in single-cell repertoires

**Fig. 3:**
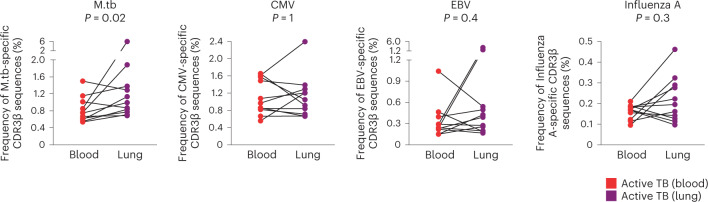
CDR3β sequences expressed on *M. tuberculosis* lysate-reactive T cells are enriched in lungs of patients with TB. Frequencies of *M. tuberculosis*- (M.tb-), CMV-, EBV- or influenza A-specific T cell clones, measured as CDR3β sequences in matched blood (PBMCs) and resected lung samples collected from people with severe active TB disease (*n* = 11). The *P* value was computed using Wilcoxon’s signed-rank test (two sided).

We then combined the CDR3β sequences obtained from mycobacteria-reactive CD4 T cells from controllers, progressors and previously published TCR datasets^13,15^, amounting to 25,256 CDR3β sequences (Supplementary Table 3). To determine whether *M. tuberculosis*-specific T cells are preferentially enriched at the site of recent or ongoing TB disease, we compared bulk TCR data generated from blood and resected lung tissue samples, collected from an independent cohort of TB patients^19^. *M. tuberculosis* lysate-reactive CD4 TCR sequences were significantly enriched in lung tissue compared with corresponding peripheral blood samples (Fig. 3). By contrast, frequencies of cytomegalovirus (CMV), Epstein–Barr virus (EBV) and influenza A-specific CDR3β sequences did not differ between blood and lung resection samples, consistent with an expansion of *M. tuberculosis*-specific TCRs at the site of recent or ongoing disease.

**Extended Data Fig. 2:**
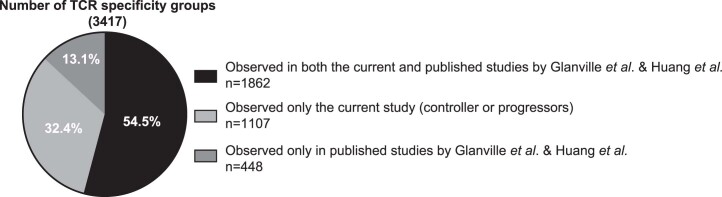
The majority of M.tb TCR specificity groups contain TCRβ sequences observed in two independent single cell TCR sorting experiments. Pie chart depicting the proportions of 3,417 M.tb TCR specificity groups, which contain TCR sequences identified by single cell TCR sequencing in both the present study and the previously published single cell TCR datasets by Glanville et al.^15^ & Huang et al.^13^ (black), or those identified only in the present study (dark grey), or reported only in the Glanville et al.^15^ & Huang et al.^13^ single cell TCR datasets (light grey).

The incredible diversity and private nature of CDR3β sequences have necessitated the development of clustering methods that group CDR3β sequences that probably share epitope specificities^13,15,16,20–22^. Such clustering methods allow interindividual comparisons of CDR3β sequences that probably share antigen specificity. We sought to determine whether such clusters of TCRs were differentially associated with either controllers or progressors. Using GLIPH2 (ref. 13) to cluster TCR-β sequences expressed by mycobacteria-reactive T cells, we identified 3,417 *M. tuberculosis* TCR similarity groups (Supplementary Table 4). Of the TCR similarity groups, 54% contained CDR3β sequences observed in sorting experiments performed in at least two independent studies^13,15^ (Extended Data Fig. 2). This observation strongly implies that most of the TCR similarity groups contained TCRs that target antigens in *M. tuberculosis* lysate.

**Fig. 4:**
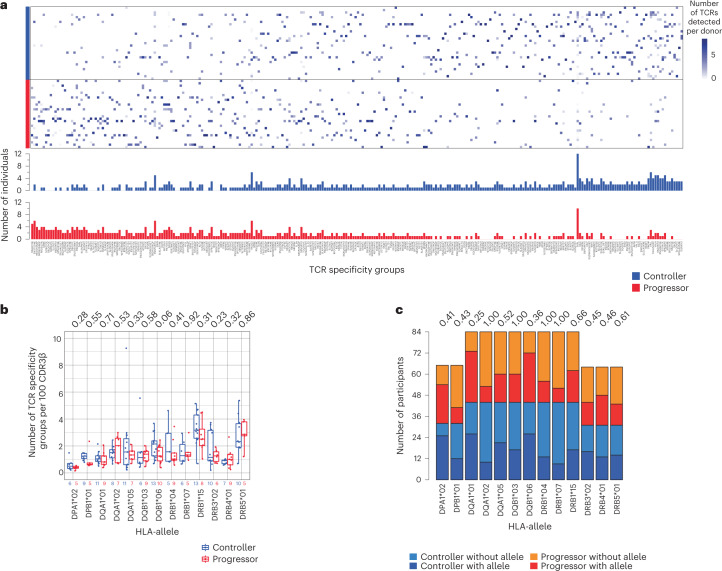
Mycobacteria-reactive TCR similarity groups overlap considerably between controllers and progressors. **a**, Heatmap depicting mycobacteria-reactive GLIPH2 TCR similarity groups (columns) identified in scTCR-seq in controllers and progressors (rows) from the ACS. The color represents the presence (blue) or absence (white) of sequences that belong to each TCR similarity group observed after *M. tuberculosis* lysate stimulation. Similarity groups are ranked according to their detected prevalence in progressors (right) or controllers (left). The barplot below depicts the number of donors possessing CDR3β sequences that belong to the indicated similarity group. The amino acid motif that is shared by TCR sequences clustered together is used to denote the cluster. In some instances GLIPH2 allows for a wildcard (that is, any amino acid) at a specific location within the shared motif; this is indicated by ‘%’. **b**, Box and whisker plots depicting the number of mycobacteria-reactive TCR similarity groups detected by scTCR-seq per 100 mycobacteria-reactive CDR3β sequences in ACS controllers and progressors. A higher value denotes greater diversity among mycobacteria-reactive CDR3β sequences. Note that not all mycobacteria-reactive CDR3β sequences fall into a similarity group. The midline represents the median, the box the interquartile range and the whiskers the 95% CI. Two-tailed Student’s *t*-test: *P* values are shown above the plot. The number of samples from controllers and progressors are indicated below each plot. **c**, Bar plots depicting the number of ACS controllers and progressors with or without the indicated HLA-allele. Fisher’s exact test (two sided) *P* values are listed above each bar.

Previously, we reported that applying filters to the GLIPH2 output parameters narrowed down the number of TCR similarity groups and enriched for groups more likely to have been clustered correctly^13^. We selected TCR similarity groups shared by three of more participants, consistent with three or more unique CDR3β sequences, with enriched common V-genes (vb_score <0.05), with a limited CDR3 length distribution (length_score <0.05) and statistically significant motifs from a reference set of CDR3β sequences (Fisher_score <0.05). This filtering resulted in 290 TCR similarity groups. We then investigated whether any of the selected TCR similarity groups were significantly enriched in sorted *M. tuberculosis* lysate-reactive CD4 T cells from controllers or progressors. Most TCR similarity groups were shared between controllers and progressors, suggesting a high degree of overlap in T cell specificities between the groups (Fig. 4a). However, the ‘S%QGTGE’ and ‘REGGTG%SP’ TCR groups appeared to be enriched in progressors (Supplementary Table 5). Although no statistically significant enrichment was observed in these single-cell analyses after multiple correction using the Benjamini–Hochberg method (*q* < 0.2), we reasoned that the low depth achieved with scTCR-seq analysis limited statistical power to detect differences in TCR repertoires between the groups.

**Extended Data Fig. 3:**
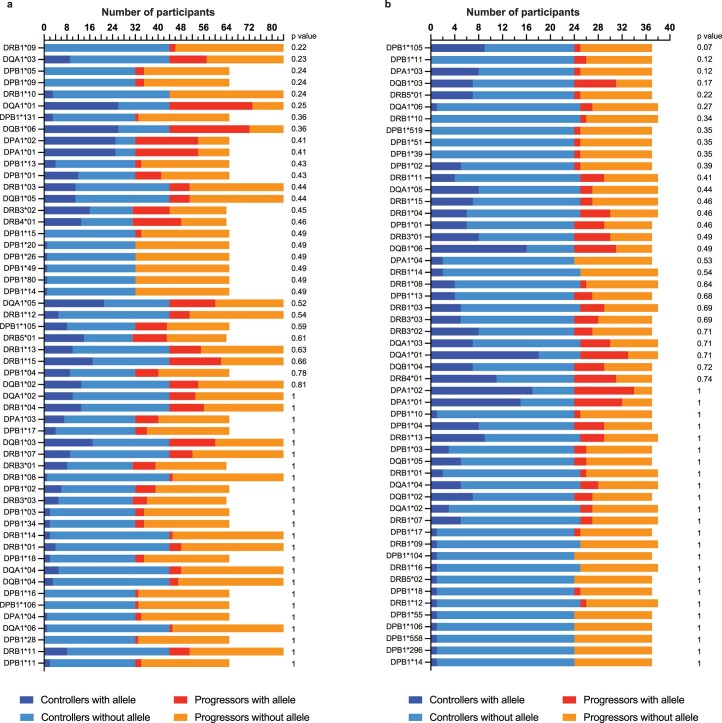
HLA allele distributions in controllers and progressors are not different. Bar plots depicting the number of controllers and progressors in the (**a**) ACS (n = 88) and (**b**) GC6 (n = 38) cohorts with or without the indicated HLA-allele. The Fisher’s exact test (two-sided) was used to compare proportions of HLA alleles between controllers or progressors. Some HLA loci have less participants because a few participants did not have complete HLA typing results at all class I and II loci.

The degree of TCR sequence diversity may be associated with control of *M. tuberculosis* or, alternatively, with progression. The large size of the *M. tuberculosis* TCR sequence dataset enabled assessment of TCR similarity group diversity within individuals. For each individual we identified the number of unique clusters with a human leukocyte antigen (HLA)-allele association identified by GLIPH2 per 100 unique CDR3β sequences. It is interesting that, among individuals with HLA-DQB1*06 alleles, we observed a trend toward increased diversity in controllers compared with progressors; however, this was not significant when we accounted for multiple testing (Fig. 4b). However, HLA-allele distribution was not associated with controller or progressor status (Fig. 4c and Extended Data Fig. 3), nor was there evidence of more allele subsets for HLA-DQB1*06 than other alleles in this population^23^, suggesting that enrichment of *M. tuberculosis* TCR similarity groups in either controllers or progressors did not simply reflect HLA-allele prevalence or allele subset diversity.

### *M. tuberculosis* TCR similarity groups associated with disease outcome

ScTCR-seq of *M. tuberculosis*-specific cells was necessary for identifying TCR similarity groups likely to target *M. tuberculosis* antigens and to identify TCR-α and TCR-β pairs that allow establishment of peptide–MHC specificity. However, scTCR-seq does not allow accurate quantification of clonotypes within the overall TCR repertoire in peripheral blood. To estimate relative frequencies of individual TCR sequences expressed by mycobacteria-reactive T cells, we performed bulk TCR-β repertoire profiling in unstimulated PBMC samples from a subset of ACS study participants (*n* = 30), who had remaining PBMC samples after single-cell sorting, and in a second longitudinal cohort of adult progressors and controllers enrolled into the Grand Challenges 6-74 (GC6-74)^24^ (Supplementary Table 1). The GC6-74 cohort comprised South African household contacts of TB patients who either developed microbiologically confirmed pulmonary TB (progressors, *n* = 12) or remained healthy (controllers, *n* = 25) (Fig. 1b). From the combined ACS and GC6-74 bulk TCR-seq data, we selected only CDR3β sequences associated with mycobacteria-reactive T cells (that is, CDR3β expressed in sorted mycobacteria-reactive T cells) (Fig. 1c).

**Fig. 5:**
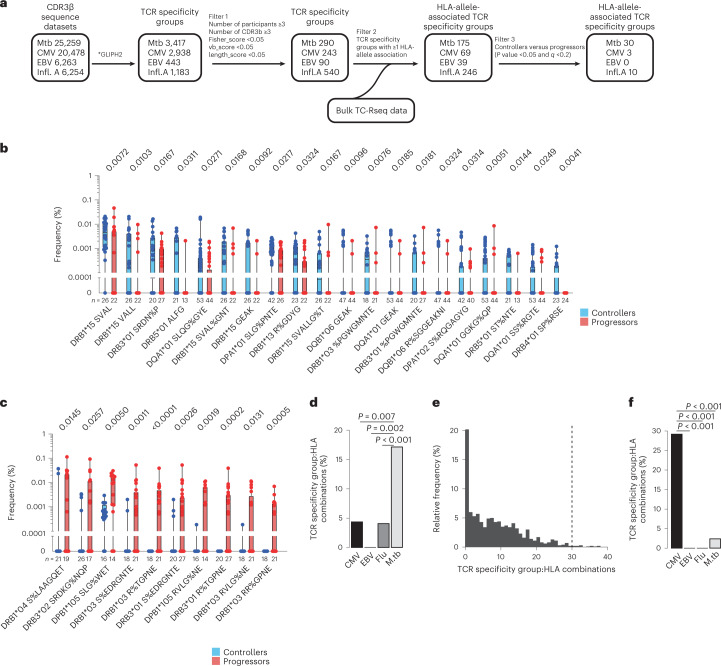
Differentially abundant mycobacteria-reactive TCR similarity groups in controllers and progressors. **a**, Analysis workflow used to measure the frequencies of mycobacteria-reactive- (Mtb-) or CMV-, EBV- or influenza A (Infl.A)-specific GLIPH2 TCR groups in controllers and progressors. GLIPH2 analysis was performed and the resulting GLIPH2 similarity groups were filtered initially using the criteria listed under Filter 1. TCR similarity groups with significant HLA-allele associations in the progressor/controller cohort were then selected (Filter 2). Similarity groups that were differentially abundant in controllers and progressors bearing the associated HLA-allele were identified (Filter 3). **b**,**c**, Box and whisker plots depicting frequencies of mycobacteria-reactive TCRs belonging to the indicated HLA-allele-associated TCR similarity groups that were significantly more abundant in controllers (**b**) or progressors (**c**) bearing the indicated HLA-allele. The horizontal lines represent medians, the boxes the interquartile range and the whiskers the range. The number of samples from controllers and progressors is indicated below each plot. Only clusters with a *P* value <0.05 (Mann–Whitney *U*-test, two sided) and *q* < 0.2 (Benjamini–Hochberg FDR) are shown. **d**, Frequencies of CMV- (3 of 69), EBV- (0 of 39), influenza A- (Flu-) (10 of 246) or *M. tuberculosis* (M.tb)-specific (30 of 175) TCR specificity group:HLA combinations that are associated with clinical outcome (significantly more abundant in either controllers or progressors), expressed as a percentage of all TCR specificity group:HLA combinations for that pathogen. The *P* value was calculated using Fisher’s exact test (two sided). **e**, Relative frequency plot of the numbers of TCR specificity group:HLA combinations found to be significantly different between the two groups. We performed permutation analyses with 1,000 iterations using randomized disease outcome labels. The vertical line represents the actual number of *M. tuberculosis*-specific TCR specificity group:HLA combinations found to be significantly different between controllers and progressors (30) with correct disease outcome labels. **f**, Frequencies of CMV- (14 of 48), EBV- (0 of 51), influenza A- (0 of 227) or *M. tuberculosis* (1 of 42)-specific TCR specificity group:HLA combinations that are associated with CMV infection status in a previously published cohort^25^, expressed as a percentage of all TCR specificity group:HLA combinations for that pathogen. The *P* value was calculated using Fisher’s exact test (two sided).

From 290 mycobacteria-reactive TCR similarity groups initially filtered on GLIPH2 output parameters, we further selected TCR similarity groups that had a significant HLA association using Fisher’s exact test *P*-value threshold of <0.05 (HLA-alleles defined by two-digit typing). Among 175 TCR similarity group:HLA-allele combinations that met this criterion, we compared frequencies of TCRs belonging to each similarity group in unstimulated PBMC samples from controllers and progressors bearing the associated HLA-allele (Fig. 5a). A total of 30 TCR similarity group:HLA-allele combinations, comprising 24 unique GLIPH2 TCR similarity groups, were differentially abundant in controllers and progressors at a *P*-value threshold <0.05, after controlling for the false discovery rate (FDR) using the Benjamini–Hochberg method (*q* < 0.2) (Fig. 5a). Twenty TCR similarity group:HLA-allele combinations had higher frequencies in controllers than progressors, whereas ten TCR similarity group:HLA-allele combinations were more abundant in progressors (Fig. 5b,c and Supplementary Table 6).

**Extended Data Fig. 4:**
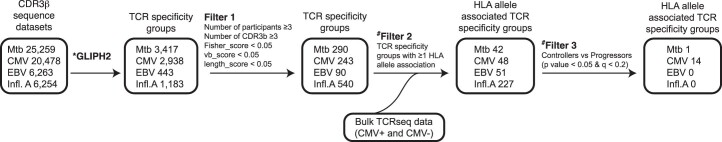
Differentially abundant CMV-specific TCR similarity groups in CMV+ persons compared to CMV- persons. Analysis workflow used to measure the frequencies of GLIPH2 TCR similarity groupings from mycobacteria-reactive (Mtb) or CMV, EBV or Influenza-A (Infl.A)-specific CDR3β sequences in CMV+ and CMV- persons. GLIPH2 analysis was performed and the resulting GLIPH2 similarity groups were filtered initially using the criteria listed under Filter 1. We then selected TCR similarity groups with significant HLA allele associations in the CMV+/CMV- cohort (Filter 2). Finally, we identified similarity groups that were differentially abundant in CMV+ and CMV- participants bearing the associated HLA allele (Filter 3).

To investigate the specificity of the disease-outcome-associated TCR similarity groups, we compared frequencies of CMV, EBV and influenza A TCR similarity groups, identified using the GLIPH2-based pipeline (Fig. 5a) in controllers and progressors. Three CMV- (4.4%, 3 of 69), zero EBV- (0%, 0 of 39) and five influenza A-specific (4.1%, 10 of 246) TCR similarity groups were differentially abundant between controllers and progressors (Fig. 5d). To test whether outcome-associated *M. tuberculosis*-reactive TCR similarity group:HLA-allele combinations were nonrandom, we performed permutation analyses using randomized disease outcome labels and determined the number of significantly associated clusters from 1,000 iterations. The 30 *M. tuberculosis*-specific GLIPH2 specificity groups associated with clinical outcome greatly exceeded the numbers obtained from 1,000 iterations with randomized disease outcome; out of the 1,000 iterations, 30 GLIPH2 specificity groups were obtained only 15 times (1.5%) (Fig. 5e). Furthermore, the number of identified CMV-, EBV- or flu-specific TCR groups fell well within the distribution obtained from the analysis with randomized outcome labels. Last, we compared the frequencies of CMV, EBV, influenza A and *M. tuberculosis* TCR similarity groups that are differentially abundant between 274 CMV-infected (CMV^+^) and 327 CMV-uninfected (CMV^−^) individuals in a bulk TCR-seq dataset published by Emerson et al.^25^ (Extended Data Fig. 4). We observed that the frequencies of 14 HLA-associated, CMV-specific TCR clusters (29%,14 of 48) were differentially abundant between CMV^+^ and CMV^−^ individuals. Thirteen clusters were significantly more abundant in CMV^+^ individuals and a single cluster was found to be more abundant in CMV^−^ individuals (Fig. 5f). By contrast, only a single HLA-associated *M. tuberculosis*-specific cluster was differentially abundant between the CMV^+^ and CMV^−^ groups and not a single EBV or influenza A-specific TCR cluster was differentially abundant in CMV^+^ and CMV^−^ individuals (Fig. 5f). Together, these results validate the specificity of our outcome-associated, *M. tuberculosis*-reactive TCR group discovery approach and suggest that the TCR groups identified were nonrandom.

**Extended Data Fig. 5:**
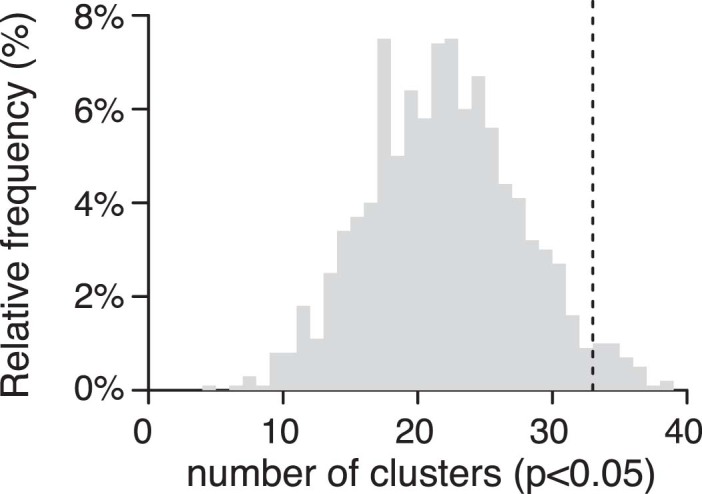
Number of TCR clusters that pass a nominal p-value threshold in permutation analysis when disease outcome label is randomized. Histogram showing the distribution of the number of TCR clusters identified at a nominal p value < 0.05 when controller and progressors status was randomized in permutation analyses with 1000 iterations. The vertical line on the right indicates the number of TCR clusters observed when the correct controller or progressor classification was used (33). 44 (4.4%) of the 1000 iterations exceeded 33.

We also used permutation analyses to further assess the robustness of our results. To do so, we first randomly permuted outcome labels 1,000×, calculating *P* values for each cluster using each set of permuted labels. From this, we calculated the distribution of counts of clusters with nominal *P* value <0.05 across the 1,000 iterations (Extended Data Fig. 5). When applying a *P*-value threshold of 0.05 to the (unpermuted, that is, original) progressor versus controller data, 33 TCR similarity group:HLA-allele combinations among the 175 were associated with outcome. Importantly, the total number of significant (*P* < 0.05) clusters exceeded 33 in only 44 of the 1,000 (4.4%) permutations, thus illustrating the presence of signal in the dataset. To identify outcome-associated clusters using a more conservative approach than the Benjamini–Hochberg method, we derived a *P*-value threshold to control the family-wise error rate at 0.05 using the permutations above. Such a threshold was made equal to the 5th percentile of the set of lowest per-permutation *P* values across the 1,000 permutations, yielding a threshold of 0.00001. None of the 175 TCR similarity group:HLA-allele combinations had an outcome-associated *P* value <0.00001 in the unpermuted progressor versus controller data, and so none was significant when controlling the family-wise error rate. We therefore controlled the less conservative FDR in our analyses.

**Extended Data Fig. 6:**
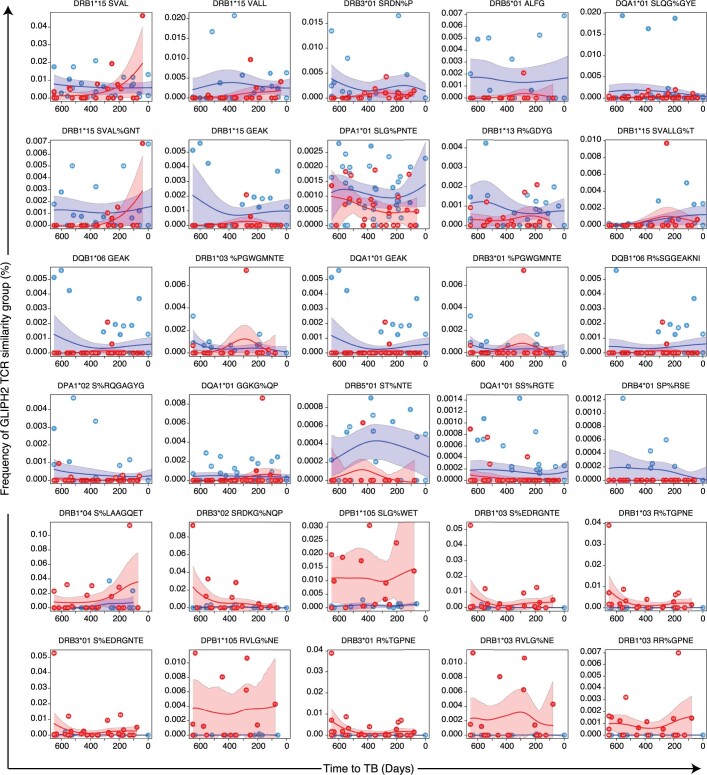
Longitudinal kinetics of differentially abundant TCR similarity clusters. Non-linear spline plots depicting the longitudinal kinetics of differentially abundant TCR similarity clusters in controllers and progressors expressing the indicated HLA allele. Samples were aligned to time to TB for progressors. For controllers, the ‘time to TB’ of their respective age-matched progressor was used. The solid lines indicate the modeled non-linear splines and the shaded bands represent 95%CI.

**Extended Data Fig. 7:**
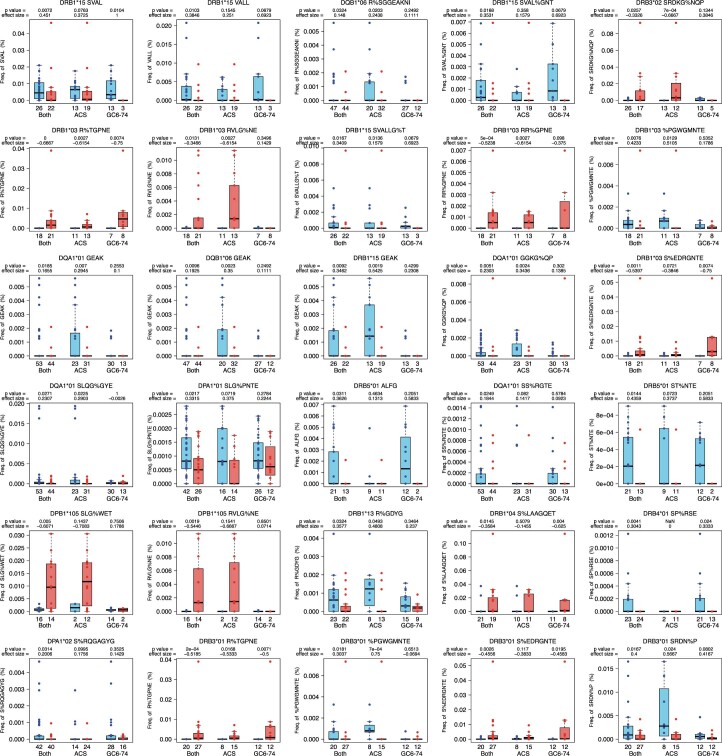
Frequencies of mycobacteria-reactive HLA-allele-associated TCR similarity groups in each of the the ACS and GC6-74 cohorts. Box and whisker plots depicting frequencies of mycobacteria-reactive TCRs belonging to the indicated HLA-allele-associated TCR similarity groups that were significantly more abundant in controllers or progressors bearing the indicated HLA-allele when data from ACS and GC6-74 participants was combined, or when ACS and GC6-74 samples were assessed separately. The horizontal lines represent medians, the boxes the interquartile range and the whiskers are the range. The number of samples from controllers and progressors are indicated below each plot. The p-value (Mann-Whitney U test, two-sided) and the effect size (Cliff’s Delta) are indicated above each plot.

We also sought to investigate the longitudinal kinetics of differentially abundant TCR similarity groups in samples collected at various timepoints before TB diagnosis in progressors, or throughout study follow-up in controllers, modeled by fitting nonlinear splines. Overall, these analyses yielded large 95% confidence intervals (CIs), highlighting the high degree of intersample and interindividual heterogeneity of *M. tuberculosis*-specific TCR data. However, the results suggest that, for many of the clusters identified to be more frequent in controllers, the TCRs were elevated in controllers throughout the study period. Similarly, TCR clusters identified as being more frequent in progressors were also generally elevated in progressors throughout the study period (Extended Data Fig. 6). To determine the influence of each cohort, we compared frequencies of the 30 differentially abundant TCR similarity group:HLA-allele combinations (Fig. 5b,c) in the ACS and GC6-74 cohorts separately. We observed concordant effect sizes between the two cohorts for most clusters, albeit with *P* > 0.05 for a number of clusters (Extended Data Fig. 7).

**Extended Data Fig. 8:**
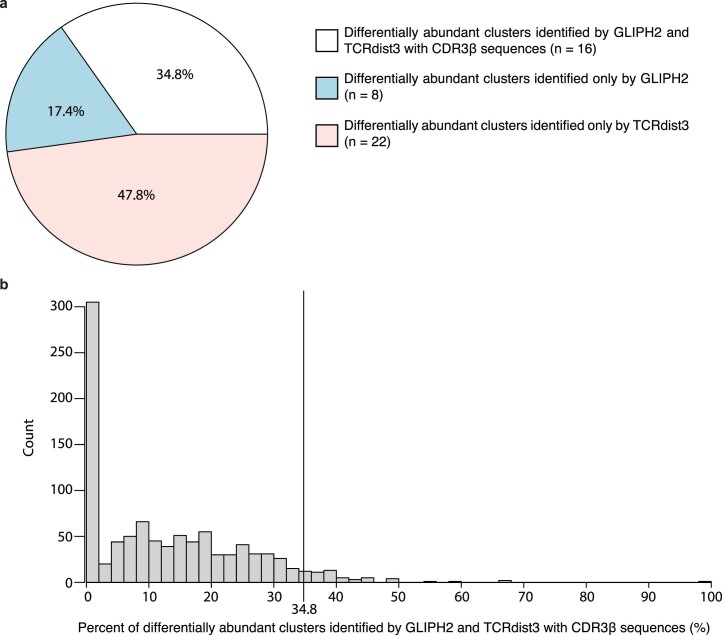
Considerable overlap between GLIPH2-identified TCR similarity clusters and TCRdist3-identified metaclone clusters found to be differentially abundant between controllers and progressors. (**a**) Pie chart showing the proportions of differentially abundant GLIPH2 similarity clusters that shared at least one CDR3β sequence with a differentially abundant TCRdist3 metaclone cluster. (**b**) Histogram showing the distribution of overlap in TCR clusters with significant association with outcome obtained from GLIPH2 or tcrdist3 when the controller and progressor status was randomized 1000 times. The vertical line on the right indicates the proportion of overlap observed when the correct classification was used (34.8%), which falls at the 94.8th percentile of the 1000 permutations.

To determine whether our results were robust to the TCR clustering algorithm, we repeated the outcome-associated TCR similarity group discovery analysis using TCRdist3 (Supplementary Table 7), another clustering algorithm^26^. The TCRdist3 pipeline identified 246 unique mycobacteria-reactive metaclone clusters with significant HLA-allele associations. Of these, 46 metaclone cluster:HLA-allele combinations consisting of 33 unique metaclone clusters were differentially abundant in controllers and progressors (Supplementary Table 8). Overall, 67% of GLIPH2-identified clusters associated with clinical outcome were also identified by TCRdist3 (16 of 24), whereas 34.8% of all clinical outcome-associated clusters identified by either GLIPH2 or TCRdist3 were identified by both (Extended Data Figure 8a). For 1,000 randomized permutations, 52 (5.2%) yielded an overlap in TCR clusters between TCRdist3 and GLIPH2 at a proportion >34.8% (Extended Data Figure 8b). Together, these data suggested that our results are largely independent of the TCR clustering algorithm.

### Identifying targets of disease-associated TCR groups

**Fig. 6:**
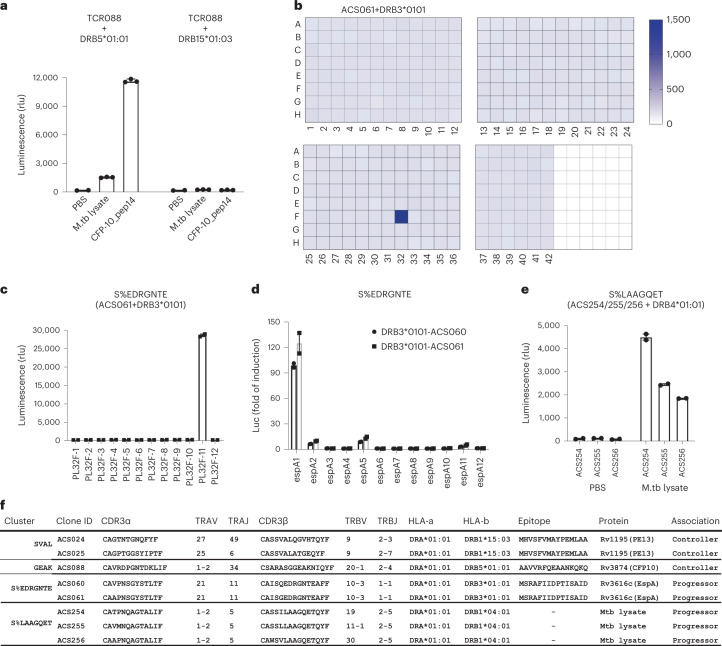
Antigen discovery for mycobacteria-reactive TCR similarity groups. **a**, Barplot showing the median relative luminescence signal after an 8-h PBS, *M. tuberculosis* (M.tb) lysate or AAVVRFQEAANKQKQ (CFP-10_p14) stimulation of TCR-ACS088 (TCR008) in the context of DRB5*01:01 or DRB1*15:03. The mean ± s.d. (*n* = 3 biological replicates) is shown. **b**, Antigen recognition screening of the whole *M. tuberculosis* proteome (321 subpools displayed in 3.5 plates) by TCR-transfected clone TCR-ACS061, bearing a TCR in similarity group S%EDRGNTE. The color scale indicates the relative luminescence signal after an 8-h stimulation of the clone. **c**, Barplot showing the deconvolution of recognition of the individual proteins from the positive subpool (PL32F), expressed separately and screened against clone TCR-ACS061. The clone was activated by PL32-F11 (Rv3616c), indicating TCR-mediated recognition. The mean (*n* = 2 biological replicates) is shown. rlu, relative luminescence units. **d**, Barplot showing resolution of the Rv3616c epitope using overlapping peptides spanning Rv3616c to identify the epitope recognized by TCR-ACS061. The mean (*n* = 2 biological replicates) is shown. **e**, Barplot depicting *M. tuberculosis* lysate recognition by clones TCR-ACS0254/255/256. The bar represents the median and each closed circle represents a replicate. The mean (*n* = 2 biological replicates) is shown. **f**, Table listing mycobacteria-reactive TCR similarity groups associated with controller or progressor status and their epitope targets.

Next, we sought to identify antigens and epitopes targeted by TCRs that belong to differentially abundant GLIPH2 TCR similarity groups (that is, similarity groups associated with either controllers or progressors). In an earlier study^15^ we observed that TCRs within the SVAL TCR similarity group targeted an Rv1195c (PE13) epitope, restricted by DRB1*15:03, and did not attempt to resolve targets for this TCR similarity group in the present study. Previously, we had also observed that TCRs within the GEAK TCR similarity group recognized an epitope that maps to Rv3874 (CFP-10), restricted by DRB5*01:01 (ref.^15^). In the present study, we observed that controllers who possessed DRB1*15 alleles had a higher frequency of the GEAK similarity cluster compared with progressors with DRB1*15. However, we did not observe activation of GEAK TCR-expressing Jurkat T cell clones in the context of DRB1*15:03, but did confirm that Rv3874 was recognized in the context of DRB5*01:01 (Fig. 6a). It is possible that other HLA-alleles in addition to DRB5*01:01 can present the CFP-10 epitope targeted by GEAK TCRs. Therefore, the association of DRB1*15 controllers with higher frequencies of GEAK TCRs may reflect CFP-10 recognition via other HLA-alleles.

**Extended Data Fig. 9:**
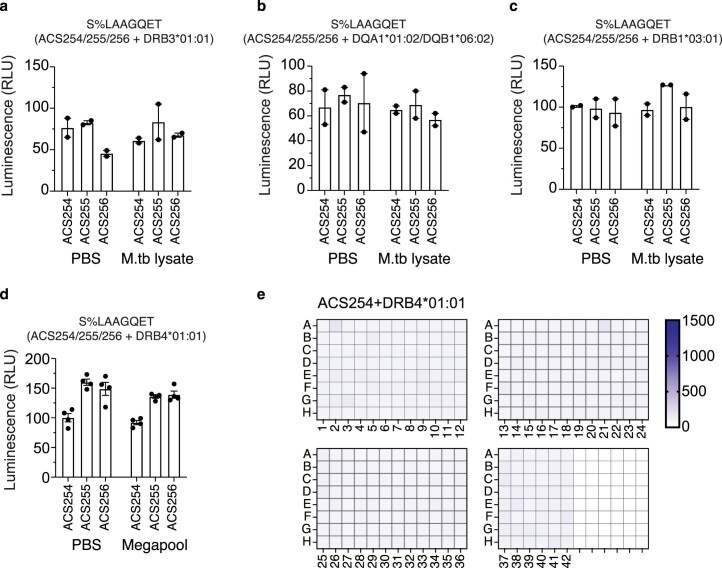
S%LAAGQET antigen discovery screen. Barplot showing the relative luminescence signal after an 8-hour PBS or M.tb lysate stimulation of TCR-ACS254, ACS255, or ACS256 in the context of (**a**) DRA*01:01/DRB3*01:01, (**b**) DQA1*01:02/DQB1*06:02 or (**c**) DRA*01:01/DRB1*03:01. The mean and range (n = 2 biological replicates) is shown (**d**) Barplot showing the relative luminescence signal after an 8-hour PBS or Mtb300 megapool stimulation of TCR-ACS254, ACS255, or ACS256 in the context DRA*01:01/DRB4*01:01. The mean and SEM (n = 4 biological replicates) is shown (**e**) Antigen screening of the whole M.tb proteome (321 subpools displayed in 3.5 plates) by TCR-transfected clone TCR-ACS254. Color scale indicates the relative luminescence signal after 8-hour stimulation of the clone.

The target of the S%EDRGNTE TCR similarity group was resolved to be a Rv3616c (EspA) epitope, restricted by DRB3*01:01 (Fig. 6b–d). We also observed that Jurkat T cell clones expressing TCR sequences in the S%LAAGQET cluster were activated by *M. tuberculosis* lysate in the context of DRB1*04:01 (Fig. 6e), but not in the context of other HLA-alleles tested (Extended Data Fig. 9a–c). We were not able to resolve the antigen/epitope target of the S%LAAGQET cluster after stimulation with the *M. tuberculosis* 300 megapool or *M. tuberculosis* protein screening library (Extended Data Fig. 9d,e). Overall, we were able to determine the antigen targets of TCRs belonging to two controller-associated TCR similarity groups and one similarity group associated with progressors (Fig. 6f).

**Extended Data Fig. 10:**
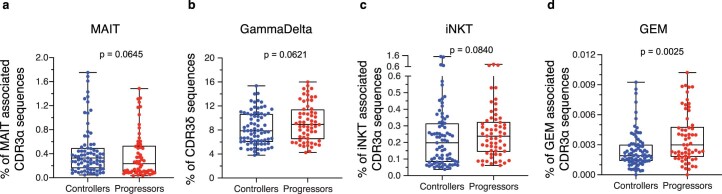
Frequencies of donor unrestricted T (DURT) cells in controllers and progressors. Box and whisker plot showing frequencies of (a) mucosal associated invariant T (MAIT) cells, (b) γδ T cells, (c), germline-encoded mycolyl lipid-reactive (GEM) T cells, and (d) invariant natural killer T (iNKT) cells, estimated from canonical TCR CDR3α sequences in PBMC samples collected from controllers (n = 77) and progressors (n = 61). Mucosal associated invariant T cells (MAIT), gammadelta, iNKT, and germline-encoded, mycolyl lipid reactive (GEM) T cells were defined as MAIT match score >=0.95, TCRDJ gene, TCRAV10;TCRAJ18 CVVSDRGSTLGRLYF, and TCRAV01-02;TCRAJ09 CAV[RL].TGGFKTIF, respectively, with ‘[]’ containing the permitted amino acid and ‘.’ denoting any amino acid. The horizontal lines represent medians, the bounds of the boxes indicate the 25% and 75% percentile and the whiskers represent the minima and maxima. Each dot represents an individual sample (controllers, n = 77; progressors, n = 61). The Mann-Whitney U test (two-sided) was used to compare frequencies between groups. P-values have not been corrected for multiple comparisons.

Last, we compared frequencies of canonical TCR CDR3α sequences of MAIT cells, CD1b-restricted, germline-encoded mycolyl lipid-reactive (GEM) cells and CD1d-restricted invariant natural killer T (iNKT) cells, as well as TCR-δ chains in ACS and GC6-74 controllers and progressors. Similar frequencies of MAIT CDR3α, iNKT and TCR-δ sequences were observed in CDR3α-sequencing data from controllers and progressors. However, progressors had higher frequencies of GEM sequences compared with controllers (Extended Data Fig. 10).

## Discussion

In the present study, we broadly surveyed CD4^+^ T cell responses to *M. tuberculosis* antigens using scTCR-seq to index TCR sequences expressed by mycobacteria-reactive T cells. We combined scTCR-seq with bulk TCR-seq and GLIPH2 analysis to identify controller-associated TCR similarity groups that may be promising targets for TB vaccine development. Traditionally, antigen discovery for vaccine development starts with the most immunogenic antigens from a given pathogen. A number of *M. tuberculosis* antigens used in candidate TB vaccines have been identified in this way^27^. However, *M. tuberculosis* expresses roughly 4,000 gene products^28^ and it remains hypothetical that the most immunogenic antigens in natural infection are the most critical immunological targets for disease control, especially as an important resistance strategy for pathogens is to avoid expulsion before transmission. By combining the power of TCR-seq and TCR analysis methods with clinically relevant cohorts, we profiled the αβ TCR response repertoire to *M. tuberculosis* between controllers and progressors without prescribing the antigens involved, and focused on those TCR specificities that associated with clinical outcome.

We successfully studied the αβ TCR repertoire of *M. tuberculosis* lysate-responsive T cells from 70 controllers or progressors of the ACS cohort, combined with single T cell data from 58 individuals previously analyzed using the same methodology, all from the broader ACS cohort. The GC6-74 cohort included 38 individuals. Thus, we analyzed the TCR-β repertoires to *M. tuberculosis* lysate of 166 *M. tuberculosis*-infected individuals and identified over 3,000 *M. tuberculosis* TCR similarity groups, a fraction that was associated with either control of *M. tuberculosis* or progression. The remainder was no different between the groups. Data in the mouse model indicate that certain T cell specificities are more important for mycobacterial control than others^29^. We therefore targeted TCR similarity groups that correlated with controllers for the identification of specific antigens that could be incorporated into a subunit vaccine, using a genome-wide antigen-screening method that we developed previously^13^, and report the identities of relevant T cell targets. This approach has applications for clinical studies of specific T cell responses to vaccination, infection and other immunological indications. Moreover, this approach represents a platform for rational antigen selection for candidate subunit vaccines that has utility for other pathogens as well.

We propose that the targets of TCR clonotype clusters associated with controllers can be considered as high-priority antigens for candidate TB subunit vaccines. Controllers possessed higher frequencies of T cells bearing PE13-specific TCRs. It is interesting that PE13 is a virulence factor that is cotranscribed with PPE18 on the same regulon under the control of Rv0485 (ref. 30). The PE and PPE family of proteins (Pro and Glu in the conserved amino-terminal region) has been implicated as key role players in host–pathogen interactions and have been investigated as potentially promising vaccine targets in murine models^31–33^. Importantly, vaccination with a PPE18 (Mtb39A)-containing polyprotein, fused with PepA (Mtb32A), showed 50% protection against TB disease in a recent landmark, phase IIb trial of the M72/AS01_E_ vaccine^34^. We also observed that controllers had higher frequencies of a TCR similarity cluster that targets a CFP-10 epitope. CFP-10 is an immunodominant antigen specific to *M. tuberculosis* and is routinely used in IFN-γ release assay (IGRA) tests to identify people infected with *M. tuberculosis*. Deleting CFP-10 and ESAT-6 from the MTBVAC vaccine, a live-attenuated TB vaccine, resulted in increased bacterial burden in the murine model^35^. Together these data suggest that further investigation of PE13 and CFP-10 as vaccine targets is warranted.

Progressors had higher frequencies of T cells bearing TCRs within the S%EDRGNTE group, which targets EspA. It is of interest that vaccination with EspA-containing subunit vaccines reduced bacterial control in mice after *M. tuberculosis* challenge^36,37^. The higher frequencies of certain T cell clones in progressors may result from clonal T cell expansion in response to increased bacterial burden during progression, as indicated by increased activation of *M. tuberculosis*-specific CD4 T cells^38^ and higher inflammation^39^ in ACS progressors than controllers. These data are therefore consistent with in vivo recognition of these antigens by T cells. It remains possible that the progression-associated T cell responses identified in the present study can also contribute to immunopathology^40–43^. This highlights the need for further assessment of T cell responses to antigens that we have identified in relevant experimental preclinical and clinical studies.

It is likely that T cell responses associated with TCR similarity groups that we observed in the present study may have been primed by Bacillus Calmette–Guérin (BCG) vaccination and/or nontuberculous mycobacteria exposure before *M. tuberculosis* infection. It is therefore difficult to determine the roles of BCG vaccination and exposure to nontuberculous mycobacteria or *M. tuberculosis* infection in driving controller-associated TCR similarity groups, although the TCR similarity group that recognizes CFP-10 is expected to be *M. tuberculosis* specific. Nevertheless, our results support the possibility that both BCG vaccination and/or *M. tuberculosis* infection may be important in driving the expansion of TCR similarity groups associated with control. For example, PE13 is expressed by both BCG and *M. tuberculosis*. Regardless of the source of priming, we propose that targeting and expanding T cell clones associated with controllers by vaccination will result in better protection from TB progression. Furthermore, we acknowledge that most CDR3β sequences were not clustered into similarity groups. Of the 16,517 unique CDR3β sequences that we observed in the mycobacteria-reactive T cell population, 5,687 unique CDR3β sequences were successfully clustered into 3,417 similarity groups. The modest proportion (34.4%) of CDR3β sequences that could be clustered together probably reflects the diversity and private nature of the TCR repertoire. We were unable to compare the frequencies of CDR3β sequences that were not clustered and therefore may have missed TCRs associated with control or progression.

We restricted analyses of associations with clinical outcome to TCR similarity groups with a significant HLA-allele association. As it is well known that HLA class II peptide binding is highly promiscuous, we expect that a nontrivial proportion of individual HLA-allele-associated T cell response differences between controllers and progressors may have been masked by this peptide–HLA promiscuity, rendering them unidentifiable with our analytical pipeline. This concept underscores the remarkable complexity and vast scope of human T cell recognition of *M. tuberculosis* proteins, as reported previously^44^, and supports future studies and orthogonal approaches to such analyses.

Our result is consistent with previous findings of no association between frequencies of T cell responses in BCG-vaccinated or MVA85A-vaccinated infants and clinical outcome^45,46^. Other T cell functions or features may be more relevant to protection. Recent data from intravenous BCG vaccination and experimental *M. tuberculosis* infection of nonhuman primates suggest that T_H_1/T_H_17 cells were associated with successful control or even sterilizing immunity^3–6,47^. Future studies that compare the differentiation state, lung homing capacity and phenotypes of antigen-specific T cells expressing controller-associated and progressor-associated TCR similarity groups may shed more light on the roles of these T cell characteristics.

Most TCR-specificity groups apparently had no association with either control or progression. We speculate that this is consistent with the hypothesis that *M. tuberculosis* allows immune recognition of considerable numbers of ‘decoy’ proteins to distract the T cell response, probably to facilitate persistence. This decoy strategy has been observed in murine studies, which showed that TB10.4 acts as a decoy antigen by inducing a TB10.4-specific CD8 T cell response that poorly recognizes infected macrophages^48,49^. This immunodominant T cell response suppressed subdominant responses and thereby subverted immune control^48,49^. A similar decoy phenomenon, involving an immunodominant epitope for CD4 T cells within the ESAT-6 protein, that subverts subdominant epitopes with greater protective capacity has also been described^50^. However, further exploration is required to adequately test this hypothesis in humans.

We acknowledge that our study has several other limitations. Our comparisons of controllers and progressors are limited to peripheral blood rather than the more relevant lung compartment^51^. It is possible that distinct T cell responses and specificities are present at sites of disease. Our study utilized samples collected exclusively from South Africans. It will be important to determine whether similar TCR similarity groups are associated with controllers from populations with different TB epidemiology, age, environmental conditions and HLA background. We also note that due to the limited sample size we restricted association analyses to two-digit HLA typing and not four-digit typing and were unable to definitively address the role of genetic variation, especially in the MHC locus, on TCR and clinical outcome between progressors and controllers. Similar larger studies using samples collected from other countries with a high TB burden will need to be performed to determine the generalizability of our results. We also note that the use of H37Rv lysate to stimulate PBMCs may have resulted in underrepresentation of TCR sequences induced by the infecting *M. tuberculosis* strain in controllers and progressors. It is not possible to identify the infecting *M. tuberculosis* strains in controllers, although the identities of *M. tuberculosis* strains in progressors were not determined. Despite these limitations, we demonstrated the utility of TCR profiling for the purpose of identifying *M. tuberculosis*-specific T cell clonotypes associated with control of *M. tuberculosis* infection and their target antigens. We note that the antigenic targets for many *M. tuberculosis* TCR similarity groups identified in the present study remain to be resolved. Regardless, the present study has provided an initial list of TCR specificities and a large TCR sequence database that can be used as a valuable tool in the search for candidate TB vaccine antigens.

## Methods

### Study populations

The ACS, including a selection of progressors and controllers (also termed nonprogressors), has been previously described^17,24,52^. Briefly, 6,363 adolescents attending high schools in the Worcester region of the Western Cape, South Africa were enrolled and followed for 24 months. Among those with evidence of *M. tuberculosis* infection, by either a positive tuberculin skin test (TST) or a QuantiFERON-TB Gold In-Tube assay (QFT, QIAGEN), 44 progressors developed microbiologically confirmed (positive by sputum smear microscopy and/or MGIT (Mycobacteria Growth Indicator Tube) liquid culture), intrathoracic disease over 2 years of follow-up. Controllers also had evidence of *M. tuberculosis* infection, but did not develop TB disease during follow-up, and were matched to progressors for age, gender, ethnicity, school of attendance and prior history of TB disease. Participants were excluded from the progressor group if they developed TB within 6 months of enrollment (or the first TST- or IGRA-positive sample) to exclude early asymptomatic disease that could have been present at the time of assessment, or if they were HIV infected. Longitudinally collected PBMC samples were available from most participants at 6-monthly intervals (Fig. 1a). A noncash voucher to the value of approximately US$7 per visit was provided to adolescent participants. This voucher could be used at a local shopping mall. The Human Research Ethics Committee of the University of Cape Town approved the study (045/2005) and all participants provided written informed assent, while parents or legal guardians provided written, informed consent. All research was performed in accordance with relevant guidelines/regulations.

The GC6-74 project has also been previously described^17,24^. HIV-uninfected, household contacts of TB cases living in Cape Town, Western Cape, South Africa were enrolled and followed for up to 2 years, with assessments at baseline, 6 months and 18 months. Progressors developed microbiologically confirmed, pulmonary TB during follow-up. Controllers did not develop TB disease during follow-up and were matched at a ratio of 2:1 to progressors. Progressors in whom TB disease developed within 3 months of baseline were excluded to avoid sampling asymptomatic disease at baseline. The TST was performed at enrollment and PBMC samples were collected and stored at enrollment, 6 months and 18 months after enrollment (Fig. 1b). As TB exposure and risk of TB are strongly associated with age, analysis included samples from GC6-74 participants aged <20 years to match the ACS cohort. GC6-74 participants were compensated for transport expenses. The Stellenbosch University Institutional Review Board (N05/11/187) approved the study. Informed consent was obtained from adults, and from minors and their parents or legal guardians.

The adult TB patient cohort has been previously described^19^. The study enrolled patients with microbiologically confirmed active or previous pulmonary TB, who underwent medically indicated lung resections to treat TB or TB sequelae. DNA was extracted from lung tissue and matched blood samples using the appropriate DNAeasy kit (QIAGEN) as per manufacturer’s instructions and subjected to bulk TCR-seq (Adaptive Biotechnologies). The Biomedical Research Ethics Committee of the University of KwaZulu-Natal approved the study (BE019/13). Written informed consent was obtained from all participants.

Finally, we utilized published CDR3β sequences generated using immunoSEQ in a CMV infection study^25^. The study used human peripheral blood samples obtained from the Fred Hutchinson Cancer Research Center Research Cell Bank biorepository of 666 healthy bone marrow donors, who underwent CMV serostatus testing. We restricted our analysis to 601 samples (274 CMV^+^ and 327 CMV^−^) where the sample metadata indicated the participant’s HLA-alleles.

### ScTCR-seq

Cryopreserved PBMCs from ACS participants were thawed, rested for 6 h and stimulated for 12 h with *M. tuberculosis* lysate (10 µg ml^−1^, BEI Resources) in the presence of anti-CD49d antibody (1 µg ml^−1^, BD Biosciences 340976) and anti-CD154-PE antibody (10 µl ml^−1^, BD Biosciences, catalog no. 555700). PBS and staphylococcal enterotoxin B (1 μg ml^−1^) were used as negative and positive controls, respectively. Samples from all study timepoints for a participant were processed on the same day. For samples with insufficient cells for a negative and/or control, only the *M. tuberculosis* lysate stimulation was performed. Next, cells were stained with LIVE/DEAD Fixable Aqua Stain (Thermo Fisher Scientific) for 30 min and, thereafter, stained with the following monoclonal antibodies for 60 min in a final volume of 50 μl: anti-CD19 (1 μl, BioLegend, catalog no. 302242), anti-CD14 (1 μl, BioLegend, catalog no. 301842), anti-CD3 (2 μl, BD Biosciences, catalog no. 563800), anti-CD4 (3 μl, BioLegend, catalog no. 300556), anti-CD8 (1 μl, BD Biosciences, catalog no. 561453), anti-TCR-αβ (2 μl, BD Biosciences, catalog no. 306720), anti-CD26 (1 μl, BioLegend, catalog no. 302704), anti-HLA-DR (1 μl, BioLegend, catalog no. 307636), anti-CD69 (0.2 μl, BD Biosciences, catalog no. 340560), anti-CD137 (0.5 μl, BD Biosciences, catalog no. 740798) and BD Horizon Brilliant Stain Buffer (37.3 μl, BD Biosciences, catalog no. 563794). Reverse transcription (RT) and sequence-specific amplification were performed in a series of three nested PCR analyses before sequencing on a MiSeq (Illumina) instrument. Single αβ^+^ T cells staining CD69^+^CD137^+^ and/or CD69^+^CD154^+^ were index sorted by FACS (BD FACS Aria-II) into 96-well plates containing 12 μl of One-Step RT-PCR buffer (QIAGEN, 9.6 μl water + 2.4 μl of 5× buffer). Next, 2 μl of phase 1 TCR primers (final concentration 0.06 μM for each C primer and 0.12 μM for each V primer), 1 μl of phase 1 phenotyping primers (final concentration 0.5 μM for each primer), 0.8 μl of enzyme mix, 0.8 μl of dNTP and 0.2 μl of molecular-grade water were added per well. Plates were then placed on a thermocycler and the following thermal profile was used to perform phase 1 PCR: (1) 36 min at 50 °C, (2) 15 min at 95 °C, (3) 30 s at 94 °C, (4) 1 min at 62 °C, (5) 1 min at 72 °C, (6) go to step (3) for 25 cycles, (7) 5 min at 72 °C and (8) hold at 4 °C.

Next, separate 96-well plates were prepared for phase 2 PCR. Note that phase 2 TCR and phenotype PCR occurred in separate plates. Into each well 2 μl of 10× buffer (HotStarTaq DNA Polymerase Kit, QIAGEN), 0.4 μl of dNTP mix, 0.1 μl of HotStarTaq, 2 μl of phase 2 TCR primers (final concentration 0.06 μM each for C primer and 0.12 μM each for V primer), 1 μl of phase 2 phenotyping primers (final concentration 0.5 μM for each primer) and molecular-grade water up to 19 μl were added. Then 1 μl from the phase 1 PCR was used as a template for phase 2 TCR PCR and 1 μl from the phase 1 PCR was used as a template for phase 2 phenotype PCR. Plates were then placed on a thermocycler and the following thermal profile was used to perform phase 2 PCR: (1) 15 min at 95 °C, (2) 30 s at 94 °C, (3) 30 s at 62 °C, (4) 1 min at 72 °C, (5) go to step (3) for 25 cycles, (6) 5 min at 72 °C and (7) hold at 4 °C.

Then, separate plates were prepared for phase 3 PCR (barcoding). Into each well, 2 μl of 10× buffer (HotStar HiFidelity Polymerase Kit, QIAGEN), 0.4 μl of dNTP, 0.1 μl of DNA polymerase, 0.2 μl of paired end mix (50 μM each) and 10.3 μl of molecular-grade water were added. This was followed by 3 μl of 1:300 dilution of 100-μM column BC, 1:75 dilution of 100-μM alpha column to each column, 3 μl of 1:300 dilution of 100-μM row BC to each row and 1 μl of template from phase 2 PCR. Plates were then placed on a thermocycler and the following thermal profile was used to perform phase 3 PCR (barcoding): (1) 15 min at 95 °C, (2) 30 s at 94 °C, (3) 30 s at 62 °C, (94) 1 min at 72 °C, (5) go back to step (2) for 30 cycles, (6) 5 min at 72 °C and (7) hold at 4 °C. Primer sequences can be found in Supplementary Tables 9–14. CDR3β sequences of sorted mycobacteria-reactive T cells from controllers and progressors were compiled using CDR3β sequences from mycobacteria-reactive T cells collected from healthy *M. tuberculosis*-infected adolescents from our previous studies^13,15^. CMV-, EBV- and influenza A-specific CDR3β sequences were obtained from VDJdb^53^. MAIT Match was used for classification of CDR3α as MAIT cell sequences^54^. CDR3α sequences with MAIT Match similarity score ≥0.95 were classified as MAIT cells.

### Bulk TCR-seq

Genomic DNA was extracted from unstimulated PBMCs of participants using the QIAGEN QIAamp DNA Blood Mini Kit. The immunoSEQ assay (Adaptive Biotechnologies) was performed to quantify TCR CDR3α and CDR3β sequences^55^. We also accessed a database of published CDR3β sequences obtained using the immunoSEQ assay from adults diagnosed with TB, who underwent clinically indicated lung resections^19^.

CDR3β sequences within the bulk TCR dataset that matched amino acid CDR3β sequences of sorted, antigen-specific single T cells, or which had common GLIPH2 CDR3β amino acid motifs, were classified as mycobacteria reactive.

### Clustering CDR3β sequences

TCRα and CDR3β sequences generated with single-cell sequencing from T cells stimulated in vitro with TB-specific antigens and sorted based on coexpression of CD69 and CD154 or CD69 and CD137 were included in GLIPH2 (ref. 13) and TCRdist3 (ref. 26) analyses using def ault settings. CDR3β sequences from sorted T cells activated by *M. tuberculosis* lysate stimulation from progressor and control PBMCs at any of the study timepoints and sorted T cells activated by TB-specific antigen stimulation from healthy, *M. tuberculosis*-infected adolescents from two previous studies, also from the larger ACS^13,15^, were combined. For GLIPH2 analysis, the pooled CDR3αβ sequence list from progressors, controllers and healthy *M. tuberculosis*-infected adolescents was uploaded to the GLIPH2 server (http://50.255.35.37:8080). We selected GLIPH2 similarity clusters that consisted of three or more unique CDR3 sequences and were present in three or more participants, with a Fisher_score ≤0.05, vb_score ≤0.05 and length_score ≤0.05. Among the identified GLIPH2 similarity clusters, we identified those with significant HLA-allele associations (at the level of two-digit HLA typing), using Fisher’s exact test at ≤0.05 (Fig. 5a). Among GLIPH2 similarity cluster:HLA combinations, we then identified those with differentially abundant TCR sequences between controllers and progressors at a *P*-value threshold <0.05 and Benjamini–Hochberg FDR *q* < 0.2.

To explore the specificity of the results obtained from the differential abundance analysis of *M. tuberculosis*-reactive TCR clusters, we performed permutation analyses using randomized disease outcome labels and determined the number of significantly associated clusters from 1,000 iterations.

To identify metaclonotypes using TCRdist3, we used a script published by Mayer-Blackwell et al.^26^ (https://tcrdist3.readthedocs.io/en/latest/public.html). We applied TCRdist3 analysis to the combined CDR3β sequence from progressors, controllers and healthy *M. tuberculosis*-infected adolescents following the same pipeline as used for GLIPH2 (shown in Fig. 3a).

### Nonlinear spline analysis

Nonlinear spline analysis of longitudinal background subtracted frequencies of T cells coexpressing CD69 and CD154 or CD69 and CD137 and differentially abundant *M. tuberculosis*-reative TCR clusters was performed using the smooth.spline function in R with four degrees of freedom; 2,000 iterations were performed to compute the 95% CIs.

### Cell culture and cell lines

Candidate TCR-α and -β chains were transduced into the nuclear factor of activated T cells (NFAT) reporter-stable J76-NFATRE-luc T cell line, which is deficient for both TCR-α and TCR-β chains^13^. Candidate HLA-alleles were individually transduced into artificial antigen-presenting cells (aAPCs), which were constructed using lentiviral transduction of CD80 and HLA-DM molecules into K562 cells.

### Antigens

*M. tuberculosis* whole-cell lysate (strain H37Rv) and *M. tuberculosis* gateway clone set (plates 1–42) were kindly provided by BEI Resources. For the whole-proteome production, every 12 open read frame clones from each plate row were pooled together as a subpool and expressed using the Expressway Cell-Free Expression System^13^. Megapool peptides, containing 300 epitopes from 90 *M. tuberculosis* proteins, were kindly provided by A. Sette (La Jolla Institute for Allergy & Immunology). For epitope screening of each identified protein, overlapping peptide libraries were purchased from Elim Biopharm.

### Antigen screen

For protein stimulation, 50 μl of aAPCs (10^6^ per ml) was preloaded with the Expressway product mixture and individual proteins in a range of 10–10,000 dilutions or protein subpools at 10-fold dilution, for 3 h at 37 °C in the standard cell culture medium. Then, 50 μl of TCR-transduced J76-NFATRE-luc cells (10^6^ per ml) were added and cocultured with aAPCs for 8 h. Then cells were harvested and luciferase activity was measured using Nano-Glo Luciferase Assay (Promega). Fold induction of luciferase activity was calculated relative to unstimulated samples. For peptide stimulation, 50 μl of TCR-transduced J76-NFATRE-luc cells (10^6^ per ml) was cocultured with 50 μl of HLA-transduced K562 cells (10^6^ per ml) in a 96-well plate. A peptide pool or individual peptide was added to the well at 2 μg ml^−1^. After incubation for 8 h, cells were harvested and luciferase activity was measured.

### Statistics and reproducibility

No statistical method was used to predetermine sample size; the sample size was based on the availability of PBMC vials stored from progressors. The experiments were not randomized and the investigators were not blinded to allocation during experiments and outcome assessment. ScTCR-seq plates containing samples from five controllers and six progressors were found to have been contaminated and data from these TCR-seq plates were excluded. Bulk TCR-seq data of one sample from an ACS participant was excluded because the sample did not show strong alignment with corresponding samples from the same participant. Bulk TCR-seq data of ten samples from the GC6-74 were excluded because these samples failed quality control metrics based on sample repertoires. Six failed due to failed material transfer and four did not show strong alignment with corresponding samples from the same participant. Mycobacteria-reactive TCR similarity groups detected by scTCR-seq were compared using a two-tailed Student’s *t*-test. We used Fisher’s exact test at ≤0.05 to identify GLIPH2 similarity clusters that were HLA associated. GLIPH2 similarity cluster:HLA combinations that were differentially abundant between controllers and progressors were identified using the two-sided Mann–Whitney *U*-test at a *P*-value threshold <0.05 and Benjamini–Hochberg FDR threshold *q* < 0.2.

### Reporting summary

Further information on research design is available in the Nature Portfolio Reporting Summary linked to this article.

## Online content

Any methods, additional references, Nature Portfolio reporting summaries, source data, extended data, supplementary information, acknowledgements, peer review information; details of author contributions and competing interests; and statements of data and code availability are available at 10.1038/s41591-022-02110-9.

## Supplementary information

Reporting Summary

Supplementary Table File containing TCR and phenotype primers use to perform nested PCR analyses.

## Data Availability

The datasets and scripts to generate the manuscript figures are available at https://github.com/SATVILab/DataTidyMusvosviTCRseq. The raw bulk CDR3α and CDR3β sequence data from the ACS and GC6-74 participants are available at 10.21417/MM2022NM.

## References

[1] O’Garra A (2013). The immune response in tuberculosis. Annu. Rev. Immunol..

[2] Scriba, T. J., Coussens, A. K. & Fletcher, H. A. Human immunology of tuberculosis. *Microbiol. Spectr*. 10.1128/microbiolspec.TBTB2-0016-2016 (2017).10.1128/microbiolspec.TBTB2-0016-201628155806

[3] Cadena AM (2018). Concurrent infection with *Mycobacterium tuberculosis* confers robust protection against secondary infection in macaques. PLoS Pathog..

[4] Darrah PA (2020). Prevention of tuberculosis in macaques after intravenous BCG immunization. Nature.

[5] Gideon HP (2015). Variability in tuberculosis granuloma T cell responses exists, but a balance of pro- and anti-inflammatory cytokines is associated with sterilization. PLoS Pathog..

[6] Gideon HP (2022). Multimodal profiling of lung granulomas in macaques reveals cellular correlates of tuberculosis control. Immunity.

[7] Pai M (2016). Tuberculosis. Nat. Rev. Dis. Prim..

[8] Barry CE (2009). The spectrum of latent tuberculosis: rethinking the biology and intervention strategies. Nat. Rev. Microbiol..

[9] Davis MM, Bjorkman PJ (1988). T-cell antigen receptor genes and T-cell recognition. Nature.

[10] Sethna Z (2020). Population variability in the generation and selection of T-cell repertoires. PLoS Comput. Biol..

[11] Carlson, C. S. et al. Using synthetic templates to design an unbiased multiplex PCR assay. *Nat. Commun*. 10.1038/ncomms3680 (2013). 10.1038/ncomms3680 24157944

[12] Han A, Glanville J, Hansmann L, Davis MM (2014). Linking T-cell receptor sequence to functional phenotype at the single-cell level. Nat. Biotechnol..

[13] Huang, H., Wang, C., Rubelt, F., Scriba, T. J. & Davis, M. M. Analyzing the *Mycobacterium tuberculosis* immune response by T-cell receptor clustering with GLIPH2 and genome-wide antigen screening. *Nat. Biotechnol*. 10.1038/s41587-020-0505-4 (2020). 10.1038/s41587-020-0505-4 PMC7541396 32341563

[14] Jorgensen JL, Esser U, Fazekas de St. Groth B, Reay PA, Davis MM (1992). Mapping T-cell receptor–peptide contacts by variant peptide immunization of single-chain transgenics. Nature.

[15] Glanville J (2017). Identifying specificity groups in the T cell receptor repertoire. Nature.

[16] Dash P (2017). Quantifiable predictive features define epitope-specific T cell receptor repertoires. Nat. Publ. Gr..

[17] Zak DE (2016). A blood RNA signature for tuberculosis disease risk: a prospective cohort study. Lancet.

[18] Sharma PK (2015). High expression of CD26 accurately identifies human bacteria-reactive MR1-restricted MAIT cells. Immunology.

[19] Ogongo P (2020). Differential skewing of donor-unrestricted and γδ T cell repertoires in tuberculosis-infected human lungs. J. Clin. Invest..

[20] Chiou S-H (2021). Global analysis of shared T cell specificities in human non-small cell lung cancer enables HLA inference and antigen discovery. Immunity.

[21] Pogorelyy MV (2019). Detecting T cell receptors involved in immune responses from single repertoire snapshots. PLoS Biol..

[22] Zhang H (2020). Investigation of antigen-specific T-cell receptor clusters in human cancers. Clin. Cancer Res..

[23] Thorstenson YR (2018). Allelic resolution NGS HLA typing of class I and class II loci and haplotypes in Cape Town, South Africa. Hum. Immunol..

[24] Suliman S (2018). Four-gene pan-African blood signature predicts progression to tuberculosis. Am. J. Respir. Crit. Care Med..

[25] Emerson, R. O. et al. Immunosequencing identifies signatures of cytomegalovirus exposure history and HLA-mediated effects on the T cell repertoire. *Nat. Genet*. 10.1038/ng.3822 (2017). 10.1038/ng.3822 28369038

[26] Mayer-Blackwell K (2021). TCR meta-clonotypes for biomarker discovery with tcrdist3 enabled identification of public, HLA-restricted clusters of SARS-CoV-2 TCRs. eLife.

[27] Andersen P, Scriba TJ (2019). Moving tuberculosis vaccines from theory to practice. Nat. Rev. Immunol..

[28] Cole ST (1998). Deciphering the biology of *Mycobacterium tuberculosis* from the complete genome sequence. Nature.

[29] Bertholet S (2008). Identification of human T cell antigens for the development of vaccines against *Mycobacterium*
*tuberculosis*. J. Immunol..

[30] Goldstone RM, Goonesekera SD, Bloom BR, Sampson SL (2009). The transcriptional regulator Rv0485 modulates the expression of a PE and PPE gene pair and is required for *Mycobacterium tuberculosis*. Virulence..

[31] Brennan MJ (2021). The enigmatic PE/PPE multigene family of mycobacteria and tuberculosis vaccination. Infect. Immun..

[32] Sampson SL (2011). Mycobacterial PE/PPE proteins at the host-pathogen interface. Clin. Dev. Immunol..

[33] Qian J, Chen R, Wang H, Zhang X (2020). Role of the PE/PPE family in host–pathogen interactions and prospects for anti-tuberculosis vaccine and diagnostic tool design. Front. Cell Infect. Microbiol..

[34] Tait DR (2019). Final analysis of a trial of M72/AS01E vaccine to prevent tuberculosis. N. Engl. J. Med..

[35] Aguilo N (2017). Reactogenicity to major tuberculosis antigens absent in BCG is linked to improved protection against *Mycobacterium tuberculosis*. Nat. Commun..

[36] Aagaard C (2020). Immunization with *Mycobacterium tuberculosis*-specific antigens bypasses T cell differentiation from prior Bacillus Calmette–Guérin vaccination and improves protection in mice. J. Immunol..

[37] Woodworth JS (2021). A Mycobacterium tuberculosis-specific subunit vaccine that provides synergistic immunity upon co-administration with Bacillus Calmette-Guérin. Nat Commun.

[38] Mpande, C. A. M. et al. Antigen-specific T cell activation distinguishes between recent and remote tuberculosis infection. *Am. J. Respir. Crit. Care Med*. 10.1164/rccm.202007-2686OC (2021). 10.1164/rccm.202007-2686OC PMC8483229 33406011

[39] Scriba TJ (2017). Sequential inflammatory processes define human progression from *M. tuberculosis* infection to tuberculosis disease. PLoS Pathog..

[40] Coscolla M (2015). *M. tuberculosis* T cell epitope analysis reveals paucity of antigenic variation and identifies rare variable TB antigens. Cell Host Microbe.

[41] Kwan CK, Ernst JD (2011). HIV and tuberculosis: a deadly human syndemic. Clin. Microbiol. Rev..

[42] Elkington PT, Bateman AC, Thomas GJ, Ottensmeier CH (2018). Implications of tuberculosis reactivation after immune checkpoint inhibition. Am. J. Respir. Crit. Care Med..

[43] Comas I (2010). Human T cell epitopes of *Mycobacterium tuberculosis* are evolutionarily hyperconserved. Nat. Genet..

[44] Lindestam Arlehamn CS (2016). A quantitative analysis of complexity of human pathogen-specific CD4 T cell responses in healthy *M. tuberculosis* infected South Africans. PLoS Pathog..

[45] Kagina BM (2010). Specific T cell frequency and cytokine expression profile do not correlate with protection against tuberculosis after bacillus Calmette–Guerin vaccination of newborns. Am. J. Respir. Crit. Care Med..

[46] Tameris MD (2013). Safety and efficacy of MVA85A, a new tuberculosis vaccine, in infants previously vaccinated with BCG: a randomised, placebo-controlled phase 2b trial. Lancet.

[47] Dijkman K (2019). Prevention of tuberculosis infection and disease by local BCG in repeatedly exposed rhesus macaques. Nat. Med..

[48] Yang JD (2018). *Mycobacterium tuberculosis*-specific CD4+ and CD8+ T cells differ in their capacity to recognize infected macrophages. PLoS Pathog..

[49] Sutiwisesak R (2020). A natural polymorphism of *Mycobacterium tuberculosis* in the esxH gene disrupts immunodomination by the TB10.4-specific CD8 T cell response. PLoS Pathog..

[50] Woodworth JS (2014). Protective CD4 T cells targeting cryptic epitopes of *Mycobacterium tuberculosis* resist infection-driven terminal differentiation. J. Immunol..

[51] Ogongo P (2021). Tissue-resident-like CD4+ T cells secreting IL-17 control *Mycobacterium tuberculosis* in the human lung. J. Clin. Invest..

[52] Scriba TJ (2017). Differential recognition of *Mycobacterium tuberculosis*-specific epitopes as a function of tuberculosis disease history. Am. J. Respir. Crit. Care Med..

[53] Goncharov M (2022). VDJdb in the pandemic era: a compendium of T cell receptors specific for SARS-CoV-2. Nat. Methods.

[54] Nielsen, M. MAIT Match-1.0. https://services.healthtech.dtu.dk/service.php?MAIT_Match-1.0

[55] Robins HS (2009). Comprehensive assessment of T-cell receptor β-chain diversity in αβ T cells. Blood.

