# Supplementary material for: T cell receptor repertoires associated with control and disease progression following Mycobacterium tuberculosis infection
Source: Nat Med. 2023 Jan 5;29(1):258–69. doi: 10.1038/s41591-022-02110-9 (PMC9873565; doi:10.1038/s41591-022-02110-9)
Supplement: Supplementary file 1 — Reporting Summary [file 41591_2022_2110_MOESM1_ESM.pdf]

Corresponding author(s): Thomas J. Scriba

Last updated by author(s): Oct 20, 2022

Reporting Summary

Nature Portfolio wishes to improve the reproducibility of the work that we publish. This form provides structure for consistency and transparency in reporting. For further information on Nature Portfolio policies, see our Editorial Policies and the Editorial Policy Checklist.

Statistics

For all statistical analyses, confirm that the following items are present in the figure legend, table legend, main text, or Methods section.

n/a	Confirmed	
☐	☒	The exact sample size (*n*) for each experimental group/condition, given as a discrete number and unit of measurement
☐	☒	A statement on whether measurements were taken from distinct samples or whether the same sample was measured repeatedly
☐	☒	The statistical test(s) used AND whether they are one- or two-sided<br>*Only common tests should be described solely by name; describe more complex techniques in the Methods section.*
☒	☐	A description of all covariates tested
☐	☒	A description of any assumptions or corrections, such as tests of normality and adjustment for multiple comparisons
☐	☒	A full description of the statistical parameters including central tendency (e.g. means) or other basic estimates (e.g. regression coefficient) AND variation (e.g. standard deviation) or associated estimates of uncertainty (e.g. confidence intervals)
☐	☒	For null hypothesis testing, the test statistic (e.g. *F*, *t*, *r*) with confidence intervals, effect sizes, degrees of freedom and *P* value noted<br>*Give P values as exact values whenever suitable.*
☒	☐	For Bayesian analysis, information on the choice of priors and Markov chain Monte Carlo settings
☒	☐	For hierarchical and complex designs, identification of the appropriate level for tests and full reporting of outcomes
☒	☐	Estimates of effect sizes (e.g. Cohen's *d*, Pearson's *r*), indicating how they were calculated

Our web collection on statistics for biologists contains articles on many of the points above.

Software and code

Policy information about availability of computer code

Data collection	Single-cell TCR sequences were acquired using Illumina instrumentation and software as described in the method session.<br>The pipeline used to analyze single-cell TCR sequences in this study has been described in the previous published literature: Han, A., Nat Biotechnol 32, 684-692 (2014). Specifically, TCR V, D, and J segments were assigned by VDJFasta-1.0, in combination with blast-2.2.17, HMMER3, and Perl 5.22. Bulk TCR sequencing was performed by Adaptive Biotechnologies' immunoSEQ Technology.
Data analysis	CDR3b sequences from Mtb-reactive CD4 T cells were clustered into GLIPH specificity clusters and metaclone clusters using GLIPH version 2 (http://50.255.35.37:8080/) and tcrdist3-0.2.0 (https://tcrdist3.readthedocs.io/en/0.2.0/). Default parameters were used for GLIPH2 and tcrdist3 analysis. Flow cytometry data was analyzed using FlowJo v10. data.

For manuscripts utilizing custom algorithms or software that are central to the research but not yet described in published literature, software must be made available to editors and reviewers. We strongly encourage code deposition in a community repository (e.g. GitHub). See the Nature Portfolio guidelines for submitting code & software for further information.

Data

Policy information about availability of data

All manuscripts must include a data availability statement. This statement should provide the following information, where applicable:

- Accession codes, unique identifiers, or web links for publicly available datasets
- A description of any restrictions on data availability
- For clinical datasets or third party data, please ensure that the statement adheres to our policy

The datasets and scripts to generate the manuscript figures are available at https://github.com/SATVILab/DataTidyMusvosviTCRseq. The raw bulk CDR3 alpha and CDR3 beta sequence data from the ACS and GC6-74 participants is available at http://doi.org/10.21417/MM2022NM.

Human research participants

Policy information about studies involving human research participants and Sex and Gender in Research.

Reporting on sex and gender	Participants in the progressor and controller groups were matched for age, gender and ethnicity. Sex and gender-based analysis were not performed due to limited sample sizes. Sex and age disaggregated data is available at https://github.com/SATVILab/DataTidyMusvosviTCRseq and http://doi.org/10.21417/MM2022NM
Population characteristics	The ACS progressor and controller cohort: 44 adolescents, aged 12-18, among 6,363 adolescents, developed microbiologically-confirmed intrathoracic disease during 2 years of follow-up (Progressors). 44 adolescents who had M.tb infection, but did not develop TB disease during follow-up, and were matched to progressors for age, gender, ethnicity, school of attendance and prior history of TB disease (Controllers). The Adolescent Cohort Study was an epidemiological study that enrolled adolescents attending high schools in the Worcester region of the Western Cape, South Africa, and followed up participants for 24 months.<br><br>The GC6-74 progressor and controller cohort: 12 participants, aged 12-19 years, who developed microbiologically confirmed pulmonary TB for an HIV-uninfected, household contacts cohort were selected. 25 participants matched for age, gender, ethnicity who did not develop TB disease during follow-up were selected from this household contact cohort.<br><br>The adult TB patient cohort: 11 patients, aged 22-68 with microbiologically confirmed active or previous pulmonary TB, who underwent medically indicated lung resections to treat TB or TB sequelae were enrolled.
Recruitment	The ACS participants were part of larger epidemiological study that enrolled 6,363 adolescents attending high schools in the Worcester region of the Western Cape, South Africa, and followed for 24 months. Healthy household contacts living in Cape Town, South Africa were enrolled into the the GC6-74 study and followed up for 24 months. For both the ACS and GC6 we matched controls by age, gender, ethnicity, but there could be other factors such as force of infection that could be different between progressors and controllers that could influence our results.<br><br>The adult TB patient who underwent medically indicated lung resections to treat active TB or TB sequelae at King Dinuzulu Hospital and Inkosi Albert Luthuli Central Hospitals in Durban, KwaZulu-Natal cohort were recruited into the adult TB patient cohort. Lung tissue could only be obtained from adult who underwent medically indicated lung resections, therefore our analysis could be bias by studying a participant group with extensive disease.
Ethics oversight	For the Adolescent Cohort Study, the Human Research Ethics Committee of the University of Cape Town approved the study (045/2005) and all participants provided written informed assent, while parents or legal guardians provided written, informed consent. All research was performed in accordance with relevant guidelines/regulations.<br><br>For the GC6-74 cohort, the Stellenbosch University Institutional Review Board (N05/11/187) approved the study. Informed consent was obtained from adults. Informed assent was obtained from minors and informed consent was obtained from their parents or legal guardians.<br><br>For the adult TB patient cohort, the Biomedical Research Ethic Biomedical Research Ethics Committee (BREC) of the University of KwaZulu-Natal approved the study (BE019/13). Written informed consent was obtained from all participants.

Note that full information on the approval of the study protocol must also be provided in the manuscript.

Field-specific reporting

Please select the one below that is the best fit for your research. If you are not sure, read the appropriate sections before making your selection.

☒ Life sciences ☐ Behavioural & social sciences ☐ Ecological, evolutionary & environmental sciences

For a reference copy of the document with all sections, see nature.com/documents/nr-reporting-summary-flat.pdf

Life sciences study design

All studies must disclose on these points even when the disclosure is negative.

Sample size	The sample size was based on the availability of PBMC vials stored from progressors.
Data exclusions	Single cell TCR sequencing plates containing samples from 5 controllers and 6 progressors was found to have been contaminated and data from these plates was excluded.<br><br>Data from one sample from the ACS sample was excluded from the bulk TCR sequence database because the sample did not show strong alignment with corresponding samples from the same participant.<br><br>Ten bulk TCR sequencing samples from the GC6-74 were excluded because these samples failed QC metrics based on sample repertoires. Six failed due to failed material transfer and four samples did not show strong alignment with corresponding samples from the same participant.
Replication	Verification of reproducibility of differentially abundant M.tb TCR similarity groups between controllers and progressors could not be performed because samples from an independent cohort of controllers and progressors were not available
Randomization	Study randomization was not applicable for the study, because the progressors were identified prospectively. To limit bias, we did not randomly select controllers, but selected controllers matched to progressors for age, gender, ethnicity, and prior history of TB disease.
Blinding	Laboratory personnel were not blinded during sample processing or analysis

Reporting for specific materials, systems and methods

We require information from authors about some types of materials, experimental systems and methods used in many studies. Here, indicate whether each material, system or method listed is relevant to your study. If you are not sure if a list item applies to your research, read the appropriate section before selecting a response.

Materials & experimental systems

n/a	Involved in the study	
☐	☒	Antibodies
☐	☒	Eukaryotic cell lines
☒	☐	Palaeontology and archaeology
☒	☐	Animals and other organisms
☒	☐	Clinical data
☒	☐	Dual use research of concern

Methods

n/a	Involved in the study	
☒	☐	ChIP-seq
☐	☒	Flow cytometry
☒	☐	MRI-based neuroimaging

Antibodies

Antibodies used	anti-CD49d, BD Biosciences 340976, Clone:L25<br>anti-CD3-BV786, BD Biosciences, Cat 563800, Clone:SK7<br>anti-CD4-BV605, Biolegend, Cat 300556, Clone:RPA-T4<br>anti-CD8-Alexa Fluor 700, BD Biosciences, Cat 561453, Clone:RPA-T8<br>anti-TCRαβ-PE-Cy7, Biolegend, Cat 306720, Clone:IP26<br>anti-CD69-APC, BD Biosciences, Cat 340560, Clone:L78<br>anti-CD137-BV711, BD Biosciences, Cat 740798, Clone:4B4-1<br>anti-CD154 (CD40L)-PE, BD Biosciences, Cat 555700, Clone:TRAP1<br>anti-CD26-FITC, Biolegend, Cat 302704, Clone:BA5b<br>anti-HLA-DR-BV421, Biolegend, Cat 307636, Clone:L243<br>anti-CD14-BV510, Biolegend, Cat 301842, Clone:M5E2<br>anti-CD19-BV510, Biolegend, Cat 302242, Clone:HIB19
Validation	All monoclonal antibodies were purchased from commercial suppliers (BD Biosciences and Biolegend). The manufacturers state that these antibodies are research use only (ROU) and have been tested for flow cytometry application using human samples. We titrated all antibodies to determine the optimal staining volumes

Eukaryotic cell lines

Policy information about cell lines and Sex and Gender in Research

Cell line source(s)	The original Jurkat T cell line, K562 cell line and HEK-293T cell line were obtained from the ATCC. Jurkat 76 T-cell line, was engineered using the Jurkat T cell line from ATCC and kindly provided by Dr. Shao-An Xue (Department of Immunology,

	University College London).
Authentication	Non authenticated
Mycoplasma contamination	Negative
Commonly misidentified lines (See ICLAC register)	No commonly misidentified cell line was used.

Flow Cytometry

Plots

Confirm that:

☒ The axis labels state the marker and fluorochrome used (e.g. CD4-FITC).

☒ The axis scales are clearly visible. Include numbers along axes only for bottom left plot of group (a 'group' is an analysis of identical markers).

☒ All plots are contour plots with outliers or pseudocolor plots.

☒ A numerical value for number of cells or percentage (with statistics) is provided.

Methodology

Sample preparation	Cryopreserved PBMCs from ACS participants were thawed, rested for 6 hours and stimulated for 12 hours with M.tb lysate (10µg/mL, BEI Resources) in the presence of anti-CD49d antibody (1µg/mL) and anti-CD154-PE antibody (10µL/mL). After stimulation, cells were harvested and stained with surface markers. Dead cells were stained using LIVE/DEAD™ Fixable Aqua Dead Cell Stain Kit from Thermo Fisher scientific. Activated CD4+ and CD8+ T cells were single-cell sorted into 96-well plate for single cell TCRa/b sequencing.
Instrument	Sorting was performed on a BD FACS Aria-II (BD Biosciences)
Software	Data were analyzed using R and FlowJo v10.
Cell population abundance	The median frequencies of activated (CD69+ and CD154+ or CD69+ and CD137+) T cells in progressor and controller samples was 0.236% (range, 0.03% -1.832%) and 0.207% (range, 0.02% - 0.667%), respectively. T cells directly in in 96-well plate containing One-Step RT-PCR buffer.To determine the purity of our cell sorts we compared the expression of CD26, a marker associated with MAIT cells, on sorted cells expressing known canonical MAIT CDR3a sequences and compared this to sorted CD4 and CD8 T cells expressing other non MAIT CDR3a sequences. We observed that the expression of CD26 aligned with the T cell subset identities.
Gating strategy	A lymphocyte gate was set on Forward scatter (FSC-A) and side scatter (SSC-A), followed by a singlet gate set on FSC-A and FCS-H. A LIVE/DEAD negative, CD14 negative, and CD19 negative, CD3 positive (live T cells) gate was set, followed by ab TCR positive gate. Next CD4 positive or CD8 positive ab T cells were selected. Gate for activated population (CD69+ and CD154+ or CD69+ and CD137+) was set based on comparing negative (PBS) treatment and M.tb lysate treatment.

☒ Tick this box to confirm that a figure exemplifying the gating strategy is provided in the Supplementary Information.
